# Contributions to the knowledge of oribatid mites (Acari, Oribatida) of Indonesia. 3. The genus *Galumna* (Galumnidae) with description of a new subgenus and seven new species

**DOI:** 10.3897/zookeys.539.6541

**Published:** 2015-11-23

**Authors:** Sergey G. Ermilov, Dorothee Sandmann, Bernhard Klarner, Rahaju Widyastuti, Stefan Scheu

**Affiliations:** 1 Tyumen State University, Tyumen, Russia; 2 Georg August University Göttingen, J.F. Blumenbach Institute of Zoology and Anthropology, Göttingen, Germany; 3 Institut Pertanian Bogor, Bogor, Indonesia

**Keywords:** Oribatid mites, systematics, morphology, *Galumna*, new subgenus and species, record, fauna, Indinesia

## Abstract

Seven new species of oribatid mites of the genus *Galumna* are described from litter and soil materials of Sumatra, Indonesia. A new subgenus, Galumna (Atypicogalumna) **subgen. n.**, is proposed; it differs from all galumnid genera and subgenera by the simultaneous presence of porose areas and sacculi on the notogaster (vs. either porose areas or sacculi present). Galumna (Galumna) calva Starý, 1997 is recorded for the first time in the Oriental region, and Galumna (Galumna) sabahna Mahunka, 1995 is recorded for the first time in the Indonesian fauna.

## Introduction

This work is a part of a continuing study of the Indonesian fauna of oribatid mites (see Ermilov et al. 2015c, d), and includes data on the genus *Galumna* Heyden, 1826 (Acari, Oribatida, Galumnidae). During taxonomic identification, ten species were found belonging to four subgenera: Galumna (Atypicogalumna) subgen. n., Galumna (Galumna) Heyden, 1826, Galumna (Cosmogalumna) Aoki, 1988 and Galumna (Neogalumna) Hammer, 1973. The main goal of the paper is to present data on the specific localities, notes on new records and overall known distribution of registered taxa, and to describe and illustrate a new subgenus and seven new species.

*Galumna* is a very large genus that was proposed by Heyden (1826) with *Notaspis alatus* Hermann, 1804 as type species. The genus comprises approximately seven subgenera and 180 species (see different opinions: Subías 2004, updated 2015; Ermilov and Anichkin 2014b; Ermilov and Bayartogtokh 2015) having a cosmopolitan distribution (Subías 2004, updated 2015). The subgeneric diagnoses for Galumna (Galumna), Galumna (Cosmogalumna) and Galumna (Neogalumna) were presented by Ermilov et al. (2013), Ermilov and Corpuz-Raros (2015) and Hammer (1973), respectively. The identification keys to selective species of Galumna (Galumna) were given by Shaldybina (1975), Balogh and Balogh (2002), Weigmann (2006), Bayartogtokh and Akrami (2014), Ermilov and Anichkin (2014c) and Ermilov et al. (2015a, b); the identification keys to all species of Galumna (Cosmogalumna) and Galumna (Neogalumna) were presented by Ermilov and Corpuz-Raros (2015) and Ermilov and Anichkin (2014b), respectively.

## Material and methods

Exact collection locality and habitat are given in the respective “Material examined” section for each species.

Specimens were mounted in lactic acid on temporary cavity slides for measurement and illustration. The body length was measured in lateral view, from the tip of the rostrum to the posterior edge of the ventral plate. Notogastral width refers to the maximum width in dorsal aspect. Lengths of body setae were measured in lateral aspect. All body measurements are presented in micrometers. Formulas for leg setation are given in parentheses according to the sequence trochanter–femur–genu–tibia–tarsus (famulus included). Formulas for leg solenidia are given in square brackets according to the sequence genu–tibia–tarsus. General terminology used in this paper follows that of Grandjean (summarized by Norton and Behan-Pelletier 2009). Drawings were made with a camera lucida using a Carl Zeiss transmission light microscope “Axioskop-2 Plus”.

## Descriptions

### Galumna (Atypicogalumna) subgen. n.

Taxon classificationAnimaliaOribatidaGalumnidae

http://zoobank.org/89548A86-BC87-4288-9C4B-39FE1E4AD445

#### Type species.

Galumna (Atypicogalumna) corpuzrarosae sp. n.

#### Subgeneric diagnosis.

With main traits of the genus *Galumna* (see Ermilov et al. 2013). Notogaster with both porose areas and sacculi. Lamellar and sublamellar lines parallel, curving backwards. Body surface without sculpture and ornamentation. Adanal lyrifissures located near to anal aperture. Legs tridactylous.

#### Etymology.

The specific name *“Atypicogalumna”* refers to the presence of porose areas and sacculi on the notogaster that is unusual for Galumnidae.

#### Remarks.

Galumna (Atypicogalumna) subgen. n. differs from all genera and subgenera of the family Galumnidae by the presence of porose areas and sacculi on the notogaster (vs. either porose areas or sacculi present).

### Galumna (Atypicogalumna) corpuzrarosae sp. n.

Taxon classificationAnimaliaOribatidaGalumnidae

http://zoobank.org/D22C0050-5B3E-4218-AB18-EC4E7C8B6107

Figs 1 , 2 , 3–4 , 5–9

#### Diagnosis.

Body size: 332–365 × 232–265. Rostral and lamellar setae setiform. Interlamellar setae represented by alveoli. Bothridial setae clavate. Anterior notogastral margin developed. Four pairs of rounded porose areas and three pairs of sacculi on notogaster. Median pore and postanal porose area present.

#### Description.

*Measurements*. Body length: 332 (holotype: male), 332–365 (nine paratypes: three females and six males); notogaster width: 232 (holotype), 232–265 (nine paratypes). Without sexual dimorphism.

*Integument*. Body color light brown. Body surface, pteromorphs, genital and anal plates punctate (visible in dissected specimens), subcapitular mentum smooth. Several short longitudinal striae present in basal part of prodorsum (postero-laterally to alveoli of interlamellar setae).

*Prodorsum*. Rostrum rounded. Lamellar (*L*) and sublamellar (*S*) lines distinct. Rostral setae (*ro*, 26–28) setiform, barbed. Lamellar setae (*le*, 12–16) thin, indistinctly barbed. Interlamellar setae (*in*) represented by alveoli. Bothridial setae (*bs*, 57–61) clavate, with long stalk and shorter head rounded and barbed distally. Exobothridial setae and their alveoli absent. Porose areas *Ad* oval, transversally oriented (16–18 × 6).

*Notogaster*. Anterior notogastral margin developed. Dorsophragmata elongated longitudinally. Four pairs of porose areas rounded, with distinct margins: *Aa* (16–20) slightly larger than *A1*, *A2* and *A3* (all 12–16). Three pairs of sacculi with minute channels and small openings: *Sa* located antero-medially and nearly to *Aa*, *S2* – medially and distanced to *A2*, *S3* – medially and nearly to *A3*. Notogastral setae represented by 10 pairs of alveoli, *la* inserted posteriorly to *Aa*. Median pore present in all specimens, located between *A3*. All lyrifissures (*ia*, *im*, *ip*, *ih*, *ips*) distinct, *im* located anteriorly and nearly to *A1*. Opisthonotal gland openings located antero-laterally to *A2*.

*Gnathosoma*. Morphology of subcapitulum, palps and chelicerae typical for *Galumna* (see Engelbrecht 1969; Ermilov and Anichkin 2010). Subcapitulum size: 82–86 × 69–73. Subcapitular setae setiform, slightly barbed, *h* (6–8) shorter than *m* (10–12) and *a* (16), *a* thickest, *h* thinnest. Two pairs of adoral setae (*or*_1_, *or*_2_, 12) setiform, hook-like distally, barbed. Palps (53) with typical setation: 0–2–1–3–9(+ω). Axillary sacculi (*sac*) distinct. Chelicerae (98) with two setiform, barbed setae; *cha* (34–36) longer than *chb* (22–24). Trägårdh’s organ long, tapered.

*Epimeral and lateral podosomal regions*. Anterior tectum of epimere I smooth. Apodemes 1, 2, sejugal and 3 well visible. Setal formula: 1–0–1–2. Setae (*1a*, *3b*, *4a*, *4b*) similar in length (4), thin, smooth. Pedotecta II rounded distally in ventral view. Discidia (*dis*) triangular. Circumpedal carinae (*cp*) distinct, clearly not reaching the insertions of setae *3b*.

*Anogenital region*. Six pairs of genital (*g*_1_–*g*_3_, 8; *g*_4_–*g*_6_, 4), one pair of aggenital (*ag*, 4), two pairs of anal (*an*_1_, *an*_2_, 4) and three pairs of adanal (*ad*_1_–*ad*_3_, 4) setae thin, smooth. Three setae on anterior edge of each genital plate. Adanal setae distanced equal from each other, inserted in one diagonal row on each side of adanal region. Setae *ad*_3 _inserted laterally to adanal lyrifissures. Postanal porose area (*Ap*) elongate oval, transversally oriented (24 × 6).

*Legs*. Morphology of leg segments, setae and solenidia typical for *Galumna* (see Engelbrecht 1969; Ermilov and Anichkin 2010). Claws smooth. Formulas of leg setation and solenidia: I (1–4–3–4–20) [1–2–2], II (1–4–3–4–15) [1–1–2], III (1–2–1–3–15) [1–1–0], IV (1–2–2–3–12) [0–1–0]; homologies of setae and solenidia indicated in Table 1. Solenidion φ of tibiae IV inserted dorsally at about 2/3 length of segment.

**Figure 1.**
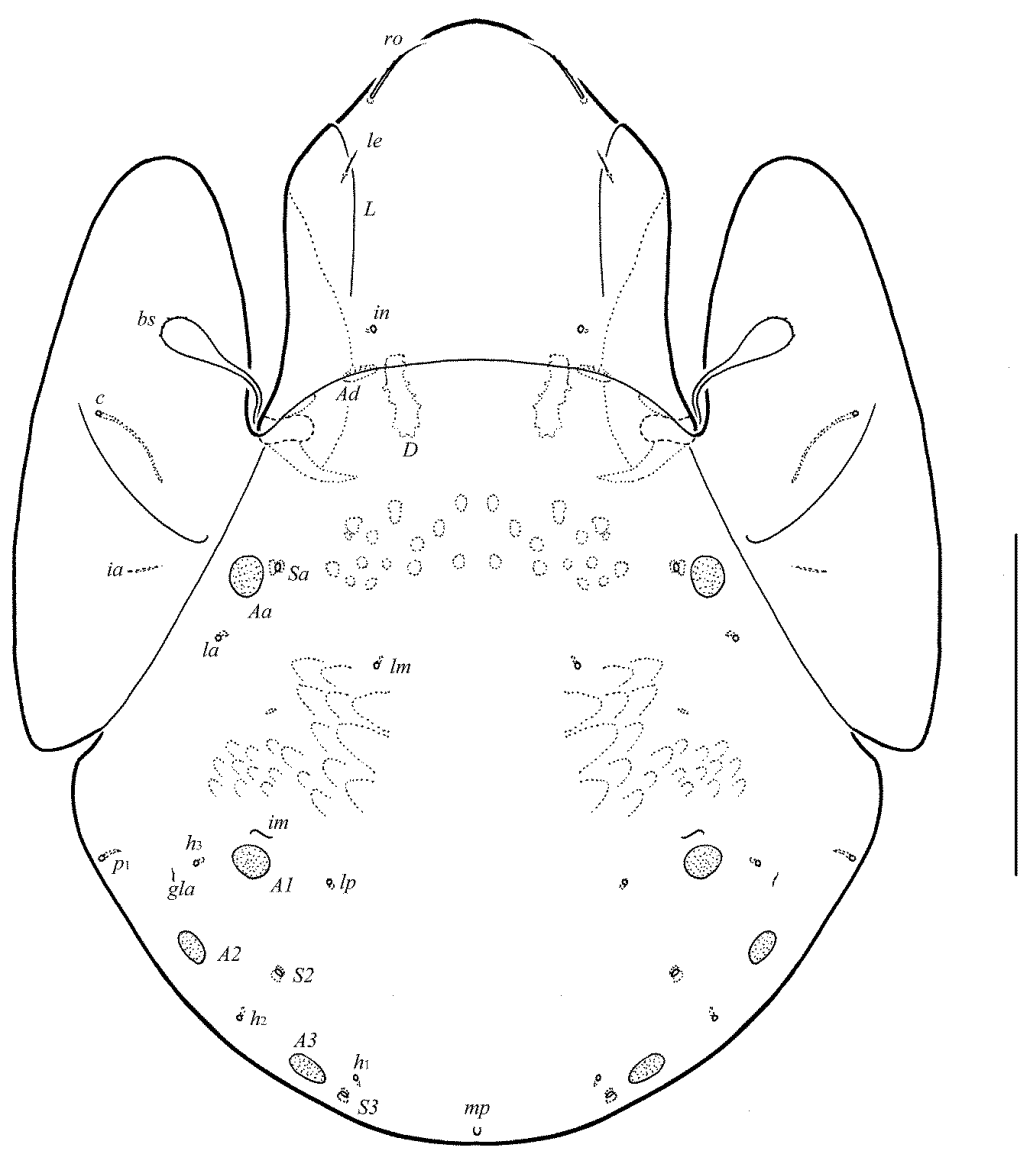
Galumna (Atypicogalumna) corpuzrarosae sp. n., adult: dorsal view. Scale bar 100 µm.

**Figure 2.**
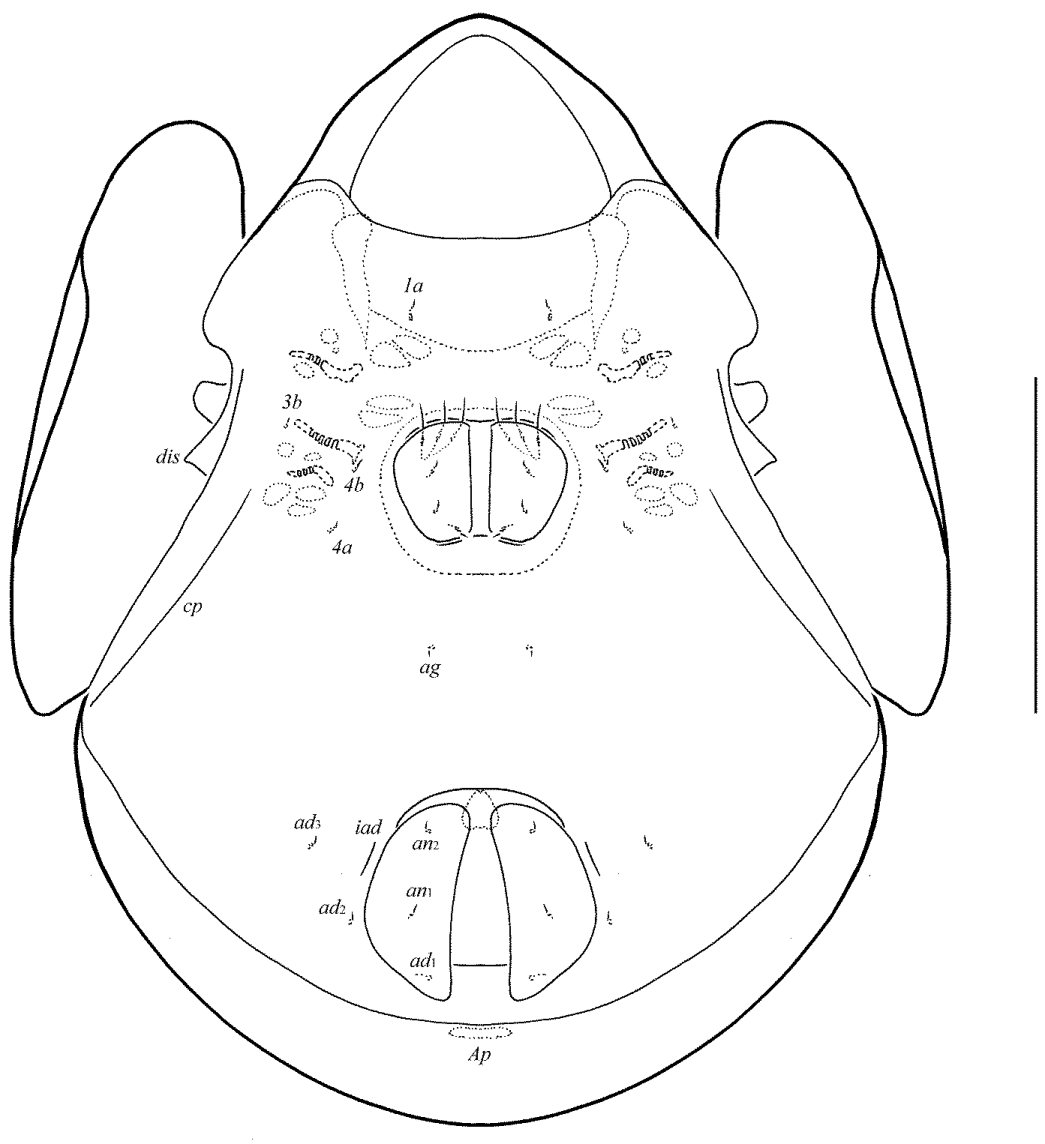
Galumna (Atypicogalumna) corpuzrarosae sp. n., adult: ventral view (gnathosoma and legs not shown). Scale bar 100 µm.

**Figures 3–4.**
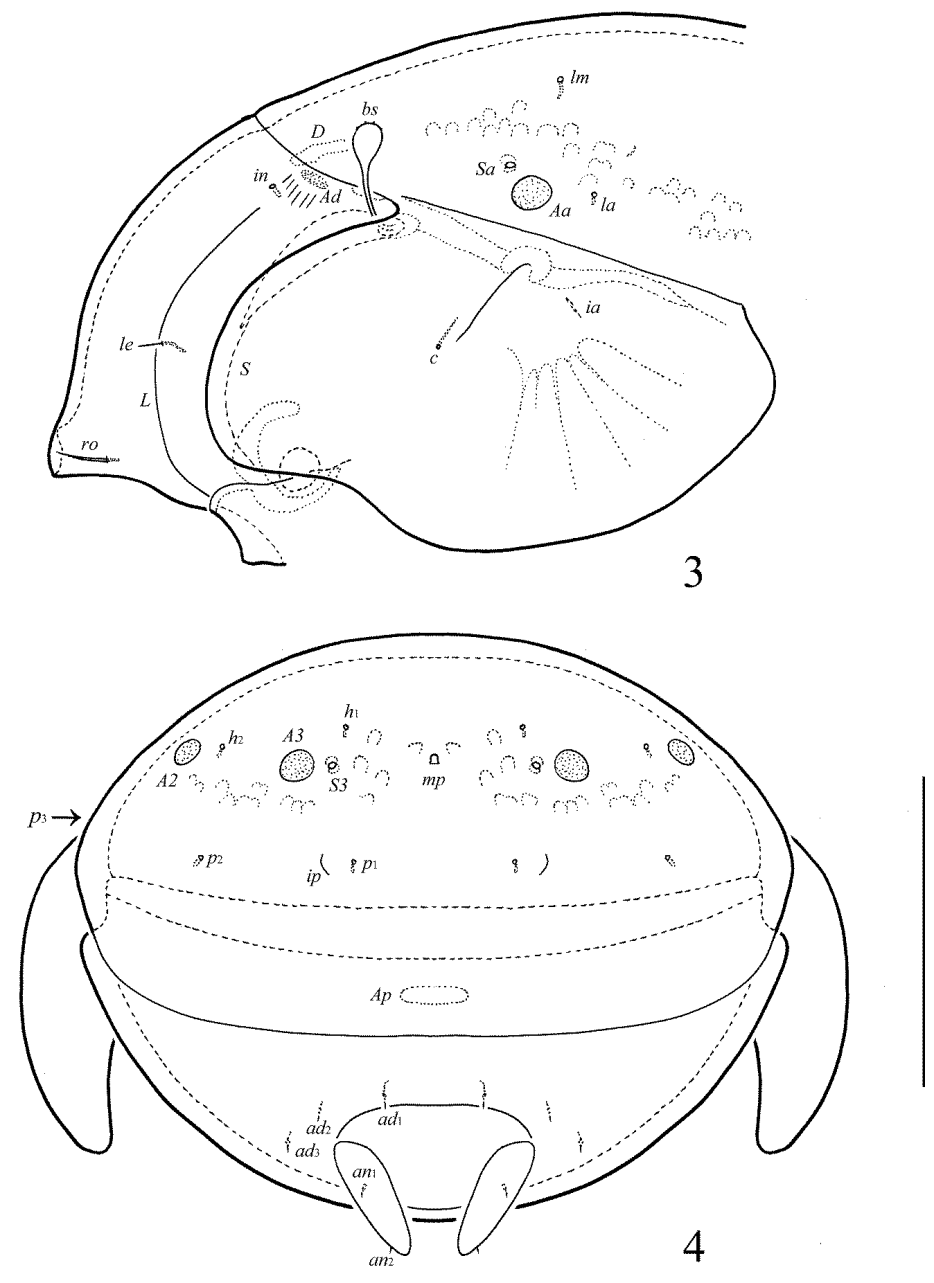
Galumna (Atypicogalumna) corpuzrarosae sp. n., adult: **3** anterior part of body, lateral view (gnathosoma and leg I not shown) **4** posterior view. Scale bar 100 µm.

**Figures 5–9.**
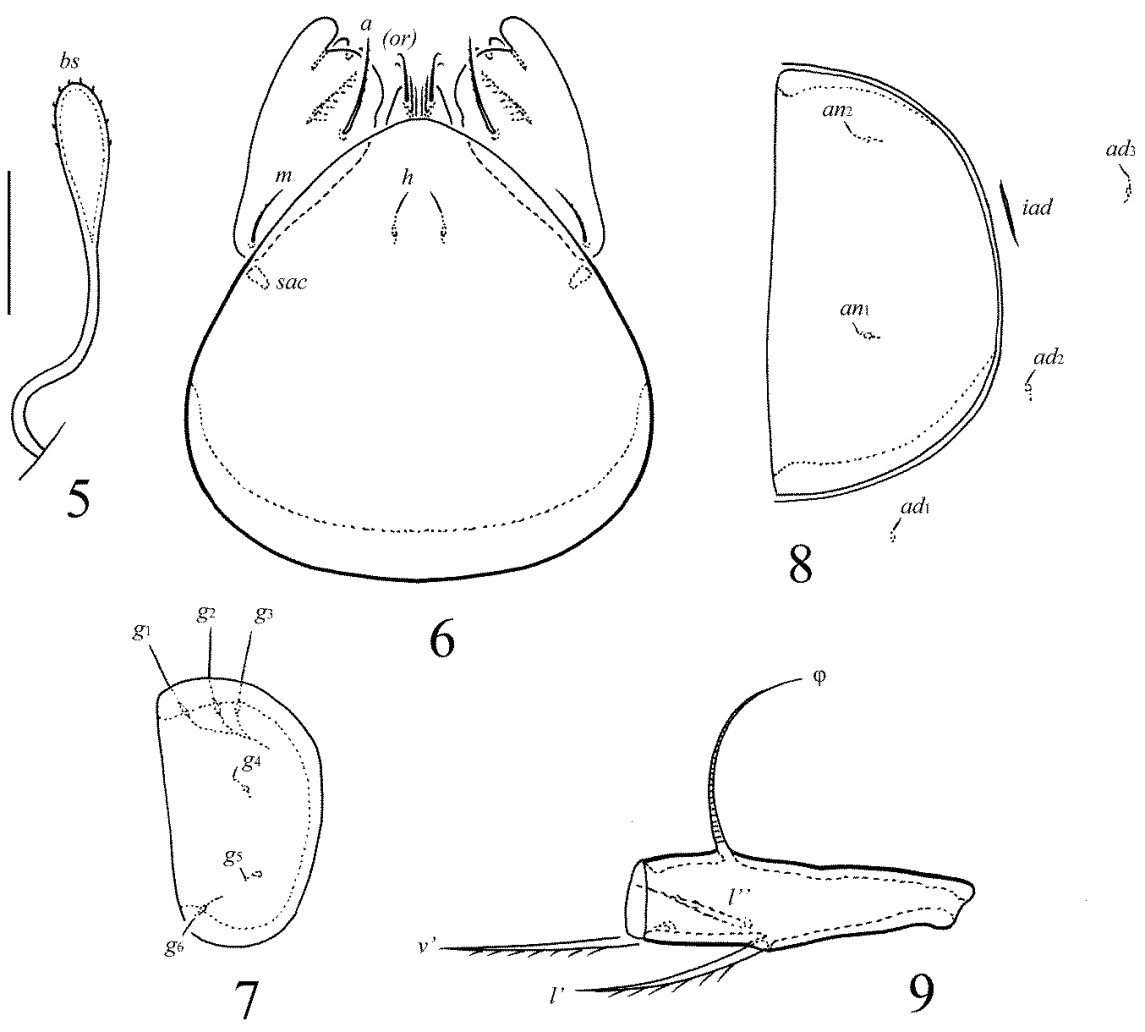
Galumna (Atypicogalumna) corpuzrarosae sp. n., adult: **5** bothridial seta **6** subcapitulum, ventral view **7** genital plate, left **8** anal plate, left, and adanal setae **9** tibia of leg IV, right, antiaxial view. Scale bar 20 µm.

**Table 1.** Leg setation and solenidia of adult Galumna (Atypicogalumna) corpuzrarosae sp. n. (same data for Galumna (Galumna) bidentatirostris sp. n., Galumna (Galumna) indonesica sp. n., Galumna (Galumna) mikoi sp. n., Galumna (Cosmogalumna) areticulata sp. n., Galumna (Cosmogalumna) sumatrensis sp. n. and Galumna (Neogalumna) specifica sp. n.)

Leg	Tr	Fe	Ge	Ti	Ta
I	*v’*	*d*, (*l*), *bv’*’	(*l*), *v’*, σ	(*l*), (*v*), φ_1_, φ_2_	(*ft*), (*tc*), (*it*), (*p*), (*u*), (*a*), *s*, (*pv*), *v’*, (*pl*), *l’*’, ε, ω_1_, ω_2_
II	*v’*	*d*, (*l*), *bv’*’	(*l*), *v’*, σ	(*l*), (*v*), φ	(*ft*), (*tc*), (*it*), (*p*), (*u*), (*a*), *s*, (*pv*), ω_1_, ω_2_
III	*v’*	*d*, *ev’*	*l’*, σ	*l’*, (*v*), φ	(*ft*), (*tc*), (*it*), (*p*), (*u*), (*a*), *s*, (*pv*)
IV	*v’*	*d*, *ev’*	*d*, *l’*	*l’*, (*v*), φ	*ft’*’, (*tc*), (*p*), (*u*), (*a*), *s*, (*pv*)

Note: Roman letters refer to normal setae, Greek letters to solenidia (except ε = famulus). Single prime (‘) marks setae on the anterior and double prime (“) setae on the posterior side of a given leg segment. Parentheses refer to a pair of setae. Tr – trochanter , Fe – femur , Ge – genu , Ti – Tibia , Ta – tarsus .

#### Material examined.

Holotype (male) and nine paratypes (three females and six males): Indonesia, Sumatra, Harapan landscape, jungle rubber agroforest, research site HJ1, 01°55'40.0"S, 103°15'33.8"E, 51 m a.s.l., in forest floor litter material. All specimens were collected by Bernhard Klarner (Nov. 2013) and identified and collected to morphospecies level by Dorothee Sandmann.

#### Type deposition.

The holotype is deposited in LIPI (Indonesian Institute of Science) Cibinong, Indonesia; six paratypes are deposited in the collection of the Senckenberg Museum, Görlitz, Germany; three paratypes are deposited in the collection of the Tyumen State University Museum of Zoology, Tyumen, Russia.

#### Etymology.

The specific name is dedicated to our friend and colleague, acarologist, Dr. Leonila Corpuz-Raros (Crop Protection Cluster, College of Agriculture and Museum of Natural History, University of the Philippines Los Baños, Los Baños, Philippines).

#### Remarks.

Galumna (Atypicogalumna) corpuzrarosae sp. n. differs from the all species of the family Galumnidae by the presence of porose areas and sacculi on the notogaster (vs. either porose areas or sacculi in other species).

### Galumna (Galumna) bidentatirostris sp. n.

Taxon classificationAnimaliaOribatidaGalumnidae

http://zoobank.org/EBA76398-87BB-4A52-A76B-45CFE76940C7

Figs 10 , 11 , 12–13 , 14–19

#### Diagnosis.

Body size: 564–664 × 448–514. Rostrum bidentate. Lamellar lines directed to lateral margins of prodorsum. Rostral setae curved medio-downwards. Lamellar setae shortest, interlamellar setae longest on prodorsum. Bothridial setae long, with dilated unilaterally, slightly barbed distally head. Anterior notogastral margin developed. Four pairs of oval porose areas on notogaster. Median pore and postanal porose area present.

#### Description.

*Measurements*. Body length: 581 (holotype: male), 564–664 (five paratypes: two females and three males); notogaster width: 464 (holotype), 448–514 (five paratypes). Without sexual dimorphism.

*Integument*. Body color brown. Body surface, pteromorphs, subcapitular mentum, genital and anal plates punctate.

*Prodorsum*. Rostrum bidentate, teeth (*t*) strong. Lamellar and sublamellar lines distinct, curving backwards, slightly divergent in distal parts, lamellar lines directed to lateral margins of prodorsum. Rostral setae (41–49) indistinctly dilated basally and curved specifically medio-downwards, smooth. Lamellar setae (24–32) setiform, thin, slightly barbed. Interlamellar setae (69–73) setiform, straight, barbed. Bothridial setae (155–176) with long, smooth stalk and short, elongated, dilated unilaterally, slightly barbed distally head. Exobothridial setae and their alveoli absent. Porose areas *Ad* oval, transversally oriented (12–16 × 4–6).

*Notogaster*. Anterior notogastral margin developed. Dorsophragmata elongated longitudinally. Four pairs of porose areas oval, with distinct margins: *Aa* (36–45 × 24–32) larger than *A1*, *A3* (32–36 × 20–24) and *A2* (16–24 × 12–20). Notogastral setae represented by 10 pairs of alveoli, *la* inserted posteriorly to *Aa*. Median pore present in all specimens, located between *A2*. All lyrifissures distinct, *im* and opisthonotal gland openings located laterally to *A1*.

*Gnathosoma*. Morphology of subcapitulum, palps and chelicerae typical for Galumna (Galumna) (see Engelbrecht 1969; Ermilov and Anichkin 2010). Subcapitulum size: 151–155 × 143–147. Subcapitular setae setiform, similar in thickness approximately, barbed, *h* (16) shorter than *m* (20–24) and *a* (24–28). Two pairs of adoral setae (16) setiform, hook-like distally, barbed. Palps (102–110) with typical setation: 0–2–1–3–9(+ω). Axillary sacculi distinct. Chelicerae (184–188) with two setiform, barbed setae; *cha* (65–69) longer than *chb* (41–45). Trägårdh’s organ long, tapered.

*Epimeral and lateral podosomal regions*. Anterior tectum of epimere I smooth. Apodemes 1, 2, sejugal and 3 well visible. Setal formula: 1–0–1–2. Setae *1a* and *3b* (24–28) setiform, barbed; *4a* and *4b* (8) thin, smooth. Pedotecta II rounded anteriorly in ventral view. Discidia triangular. Circumpedal carinae distinct, little not reaching the insertions of setae *3b*.

*Anogenital region*. Six pairs of genital (*g*_1_, *g*_2_, 14–18; *g*_3_–*g*_6_, 6–8), one pair of aggenital (8–12), two pairs of anal (8–12) and three pairs of adanal (8–12) setae thin, smooth. Two setae on anterior edge of each genital plate. Adanal setae *ad*_3 _inserted laterally to adanal lyrifissures. Postanal porose area elongated, transversally oriented (36–45 × 8–12).

*Legs*. Morphology of leg segments, setae and solenidia typical for Galumna (Galumna) (see Engelbrecht 1969; Ermilov and Anichkin 2010). Tridactylous, claws smooth. Formulas of leg setation and solenidia are similar to Galumna (Atypicogalumna) corpuzrarosae sp. n. (Table 1). Solenidion φ of tibiae IV inserted dorsally at about 2/3 length of segment.

**Figure 10.**
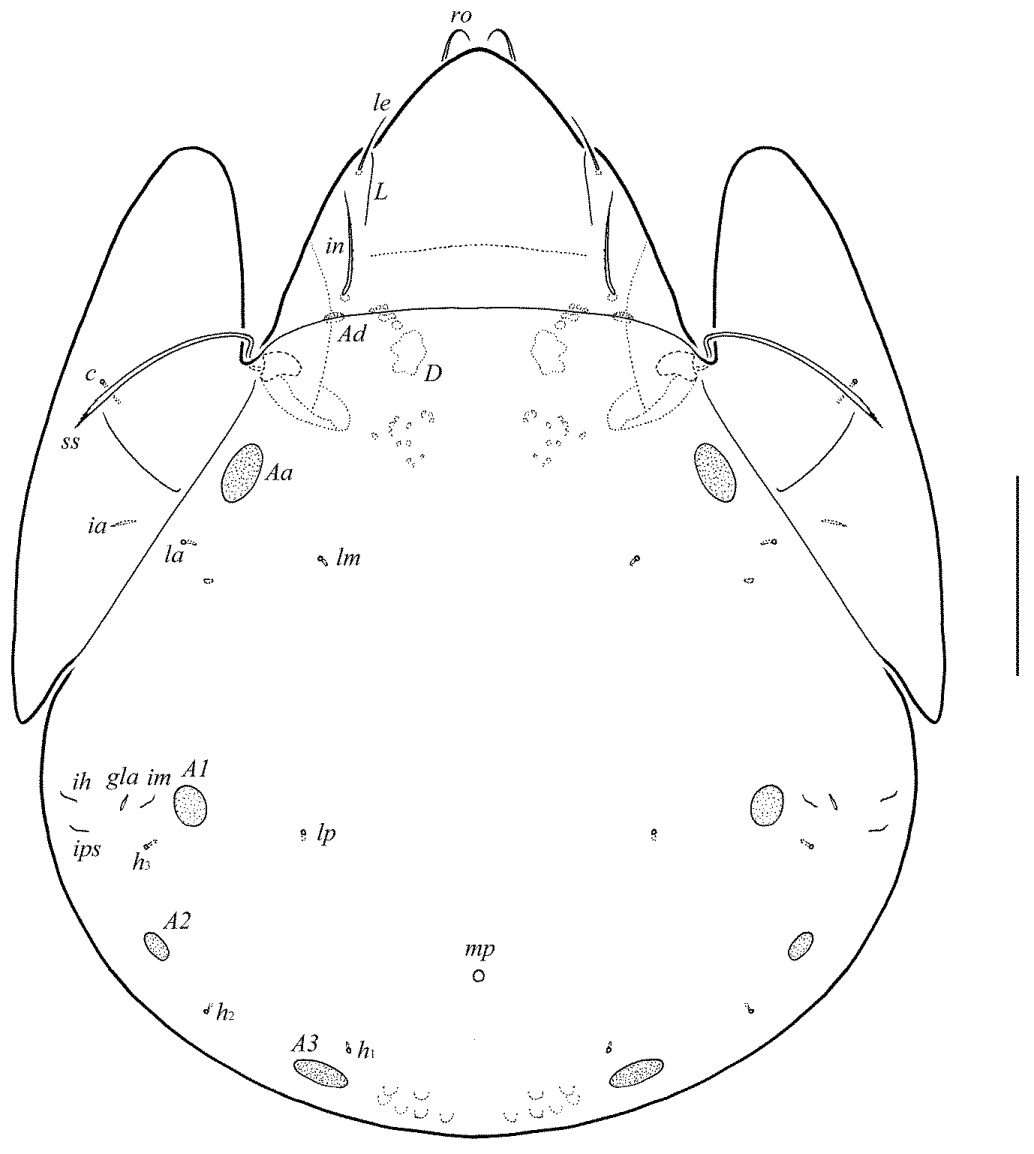
Galumna (Galumna) bidentatirostris sp. n., adult: dorsal view. Scale bar 100 µm.

**Figure 11.**
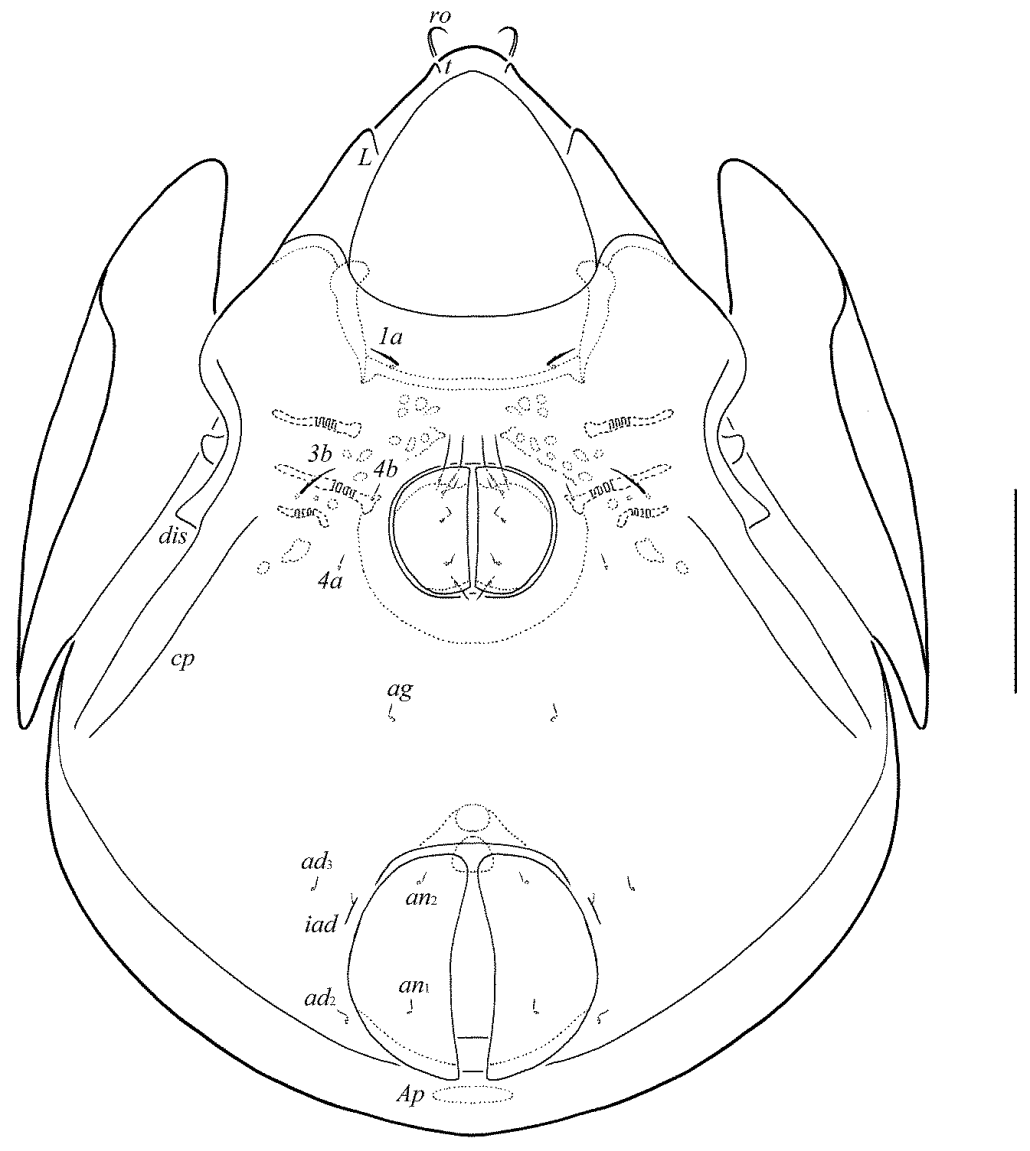
Galumna (Galumna) bidentatirostris sp. n., adult: ventral view (gnathosoma and legs not shown). Scale bar 100 µm.

**Figures 12–13.**
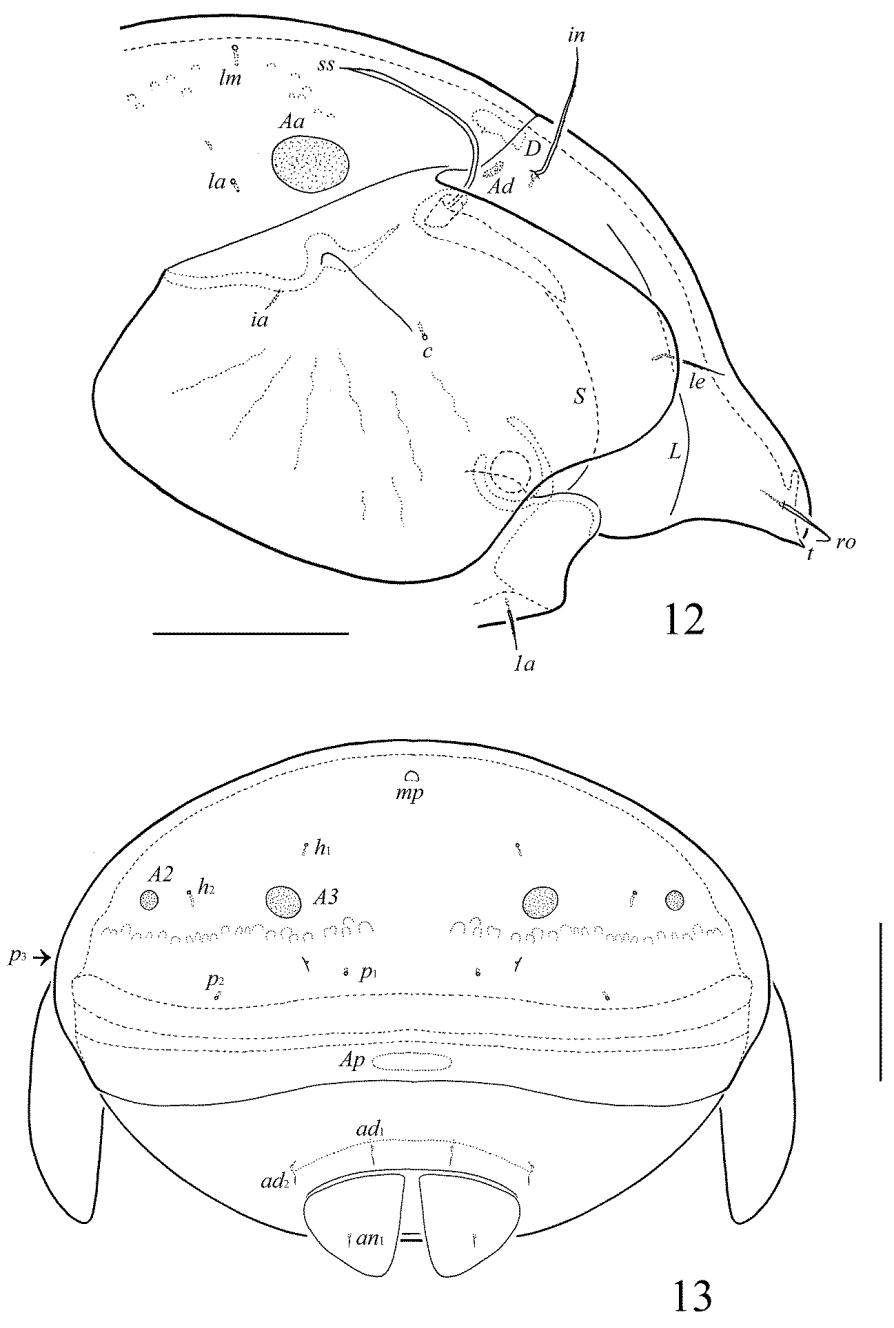
Galumna (Galumna) bidentatirostris sp. n., adult: **12** anterior part of body, lateral view (gnathosoma and leg I not shown) **13** posterior view. Scale bars 100 µm.

**Figures 14–19.**
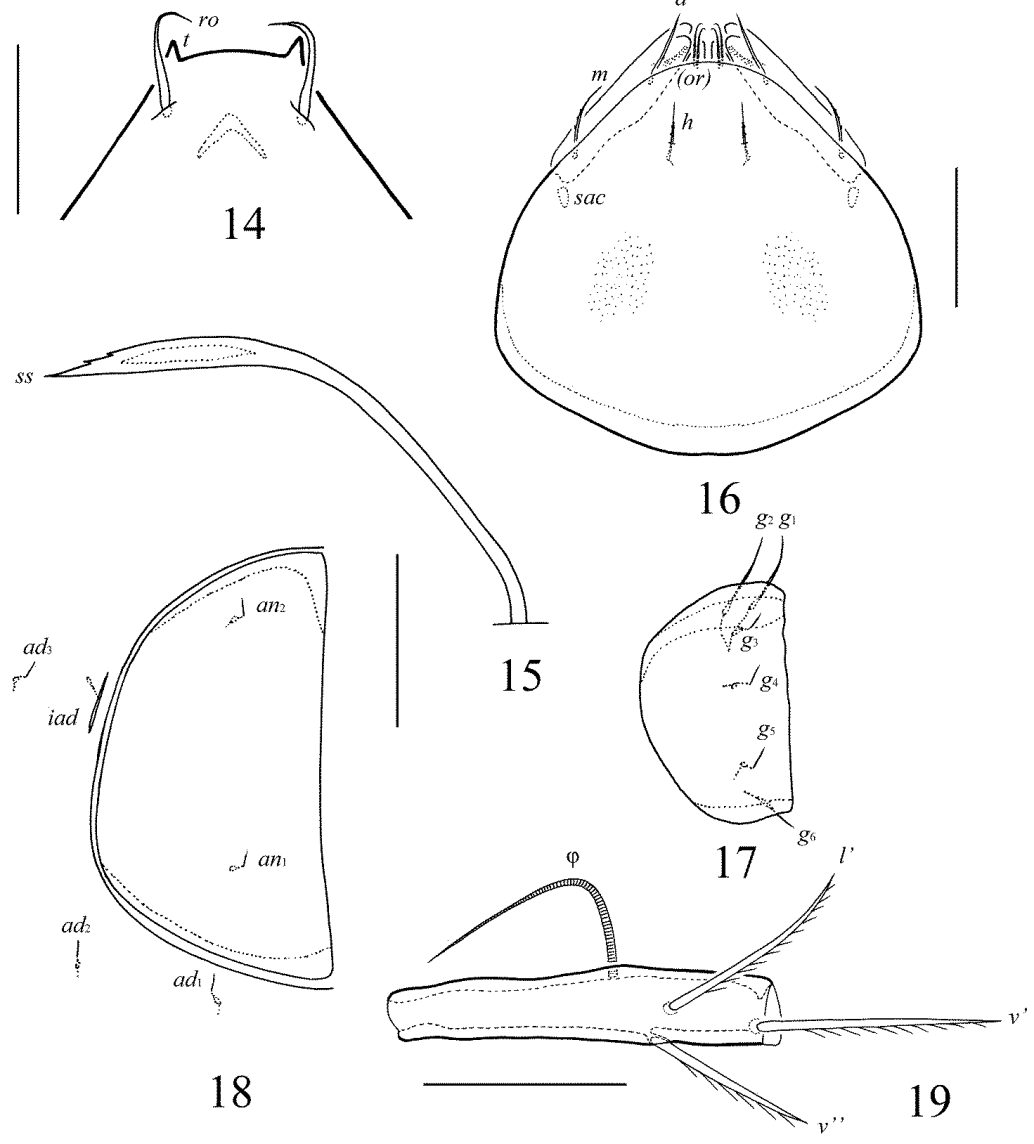
Galumna (Galumna) bidentatirostris sp. n., adult: **14** rostrum and rostral setae, dorso-frontal view **15** bothridial seta **16** subcapitulum, ventral view **17** genital plate, right **18** anal plate, right, and adanal setae **19** tibia of leg IV, left, antiaxial view. Scale bars 50 µm.

#### Material examined.

Holotype (male) and five paratypes (two females and three males): Indonesia, Sumatra, Harapan landscape, oil palm plantation, research site HO1, 01°54'35.6"S, 103°15'58.3"E, 81 m a.s.l., in upper soil layer (0–5 cm). All specimens were collected by Bernhard Klarner (Nov. 2013) and identified and collected to morphospecies level by Dorothee Sandmann.

#### Type deposition.

The holotype is deposited in LIPI (Indonesian Institute of Science) Cibinong, Indonesia; two paratypes are deposited in the collection of the Senckenberg Museum, Görlitz, Germany; three paratypes are deposited in the collection of the Tyumen State University Museum of Zoology, Tyumen, Russia.

#### Etymology.

The specific name *bidentatirostris* refers to the bidentate rostrum.

#### Remarks.

Galumna (Galumna) bidentatirostris sp. n. is morphologically most similar to Galumna (Galumna) gibbula Grandjean, 1956 from the Mediterranean (see Grandjean 1956) in having four pairs of oval porose areas on notogaster, long bothridial setae with elongated, unilaterally head, similar relative lengths of prodorsal setae (*in* > *ro* > *le*), median pore and elongated postanal porose area. However, the new species differs from the latter by the presence of bidentate rostrum (vs. without teeth in Galumna (Galumna) gibbula), rostral setae curved medio-backwards (vs. not curved in Galumna (Galumna) gibbula), anterior notogastral margin (vs. not developed in Galumna (Galumna) gibbula), lamellar lines directed to lateral margins of prodorsum (vs. directed to insertions of rostral setae), and the absence of an apophysis in the posterior part of the notogaster (vs. present in Galumna (Galumna) gibbula).

### Galumna (Galumna) indonesica sp. n.

Taxon classificationAnimaliaOribatidaGalumnidae

http://zoobank.org/508FCA63-79EB-4F0D-B909-7BD1A2EBF53A

Figs 20 , 21 , 22–23 , 24–28

#### Diagnosis.

Body size: 498–531 × 365–381. Lamellar lines straight, directed to rostrum. Prodorsal setae setiform, barbed, lamellar setae shortest, interlamellar setae longest. Bothridial setae setiform, ciliate unilaterally. Anterior notogastral margin developed. Four pairs of porose areas present on notogaster, *Aa* booth-shaped to elongate triangular, transversally oriented, *A1*, *A2* and *A3* rounded. Median pore and postanal porose area present.

#### Description.

*Measurements*. Body length: 531 (holotype: female), 498–531 (three paratypes: two females and one male); notogaster width: 381 (holotype), 365–381 (three paratypes). Without sexual dimorphism.

*Integument*. Body color brown. Body surface, pteromorphs, subcapitular mentum, genital and anal plates punctate (visible in dissected specimens).

*Prodorsum*. Rostrum rounded. Lamellar lines straight, directed little laterally to insertions of rostral setae. Sublamellar lines curving backwards. Rostral (45–49), lamellar (24–28) and interlamellar (61–73) setae setiform, barbed. Bothridial setae (106–110) long, setiform, densely ciliate unilaterally. Exobothridial setae and their alveoli absent. Porose areas *Ad* oval, transversally oriented (16–20 × 8–12).

*Notogaster*. Anterior notogastral margin developed. Dorsophragmata elongated longitudinally. Four pairs of porose areas with distinct margins: *Aa* (36–49 × 12–16) booth-shaped to elongate triangular, transversally oriented; *A1*, *A2* and *A3* (24–32) rounded. Notogastral setae represented by 10 pairs of alveoli, *la* inserted posteriorly to *Aa*. Median pore present in all specimens, located between *A2*. All lyrifissures distinct, *im* and opisthonotal gland openings located laterally to *A1*.

*Gnathosoma*. Morphology of subcapitulum, palps and chelicerae typical for Galumna (Galumna) (see Engelbrecht 1969; Ermilov and Anichkin 2010). Subcapitulum size: 118–123 × 102–106. Subcapitular setae setiform, similar in thickness approximately, barbed, *h* (18–20), *m* (20) and *a* (20–24) differ little in length. Two pairs of adoral setae (16–18) setiform, hook-like distally, barbed. Palps (82) with typical setation: 0–2–1–3–9(+ω). Axillary sacculi distinct. Chelicerae (155) with two setiform, barbed setae; *cha* (57) longer than *chb* (32). Trägårdh’s organ long, tapered.

*Epimeral and lateral podosomal regions*. Anterior tectum of epimere I smooth. Apodemes 1, 2, sejugal and 3 well visible. Setal formula: 1–0–1–2. Setae thin, smooth, *3b* (20–24) longer than *1a*, *4a* and *4b* (8). Pedotecta II rounded anteriorly in ventral view. Discidia triangular. Circumpedal carinae distinct, little, not reaching the insertions of setae *3b*.

*Anogenital region*. Six pairs of genital (*g*_1_–*g*_3_, 8–10; *g*_4_–*g*_6_, 4), one pair of aggenital (4), two pairs of anal (4) and three pairs of adanal (4) setae thin, smooth. Three setae on anterior edge of each genital plate. Adanal setae *ad*_3 _inserted laterally to adanal lyrifissures. Postanal porose area elongated, transversally oriented (32–36 × 10–16).

*Legs*. Morphology of leg segments, setae and solenidia typical for Galumna (Galumna) (see Engelbrecht 1969; Ermilov and Anichkin 2010). Tridactylous, claws smooth. Formulas of leg setation and solenidia are similar to Galumna (Atypicogalumna) corpuzrarosae sp. n. (Table 1). Solenidion φ of tibiae IV inserted dorsally at about 2/3 length of segment.

**Figure 20.**
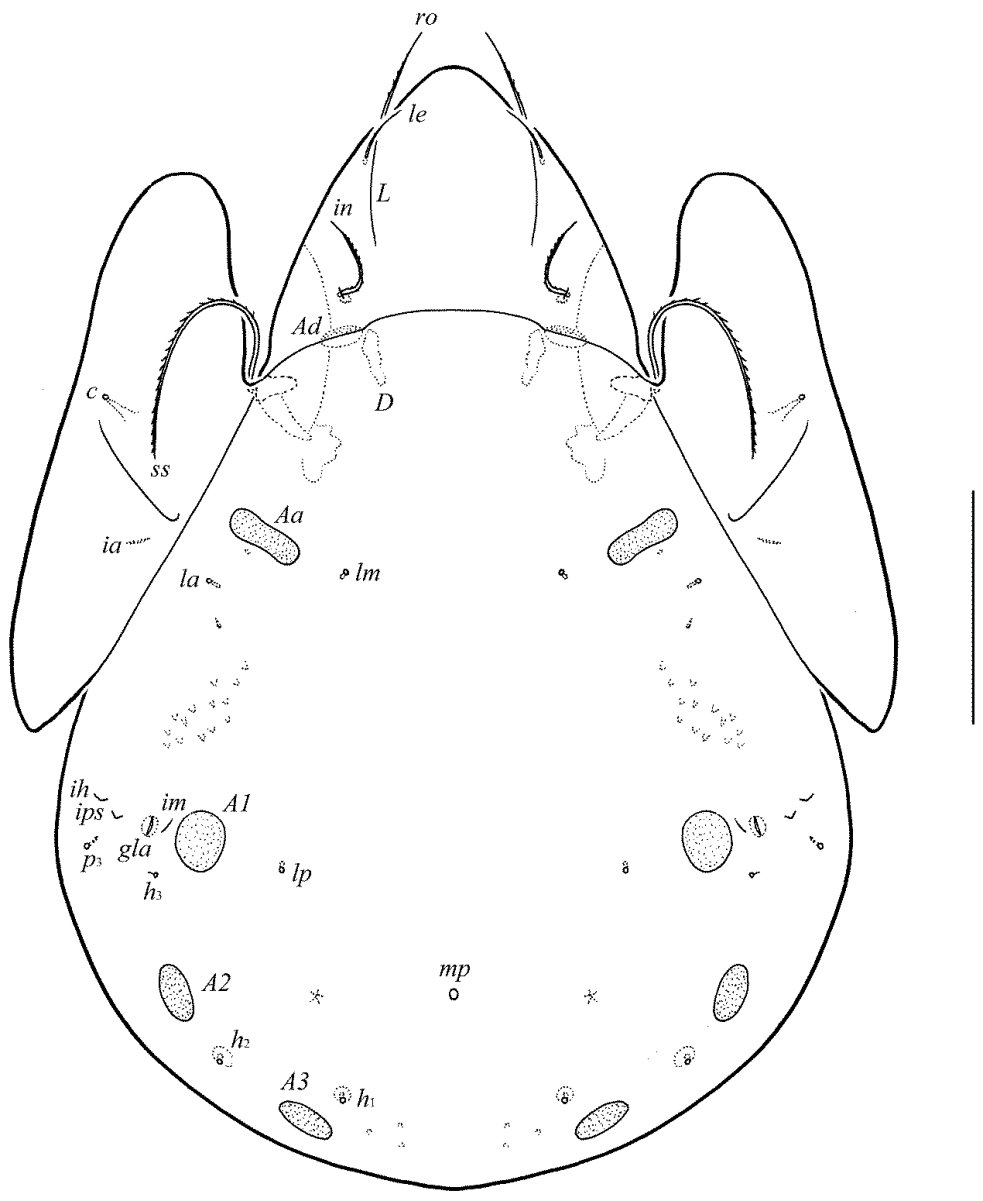
Galumna (Galumna) indonesica sp. n., adult: dorsal view. Scale bar 100 µm.

**Figure 21.**
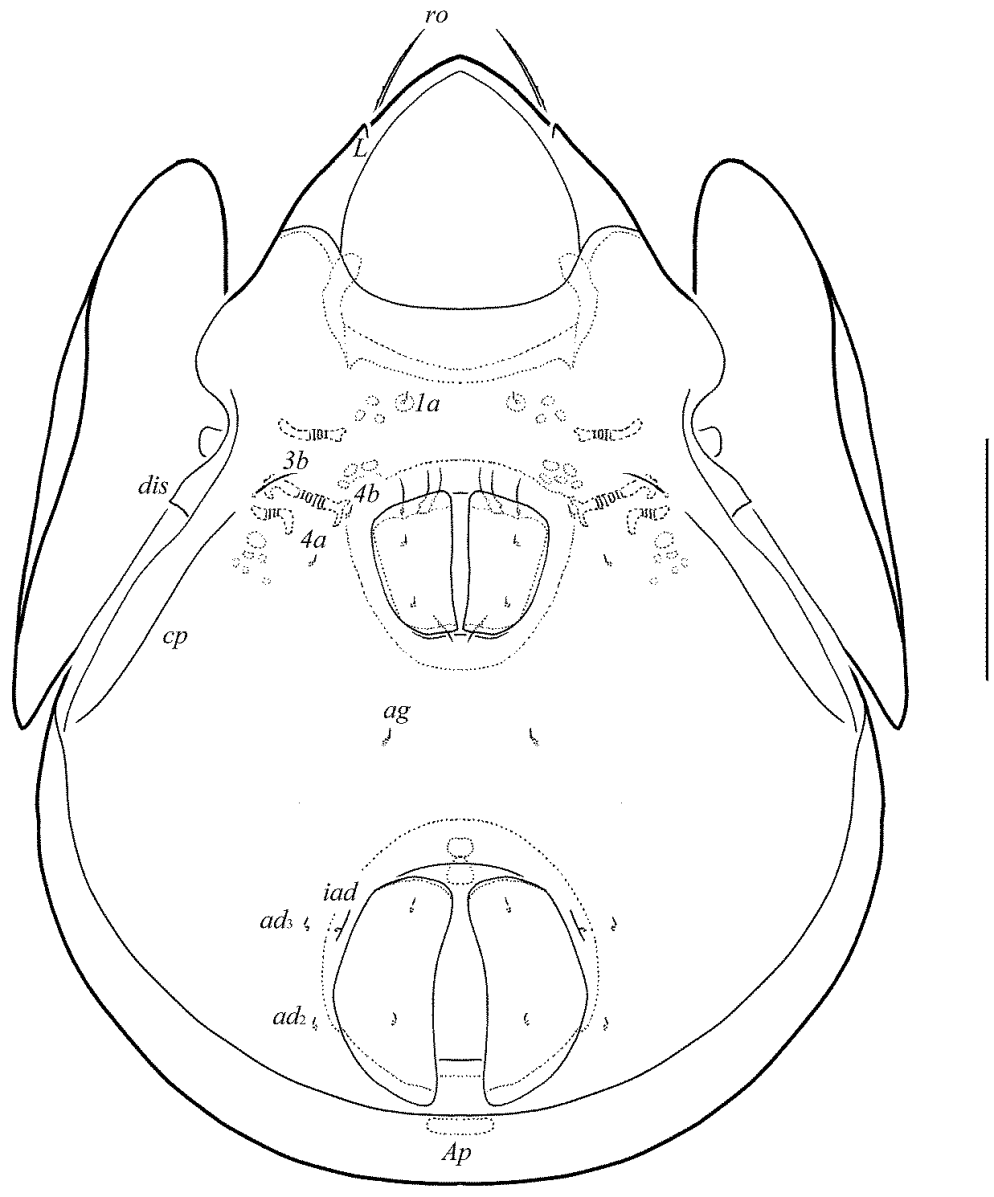
Galumna (Galumna) indonesica sp. n., adult: ventral view (gnathosoma and legs not shown). Scale bar 100 µm.

**Figures 22–23.**
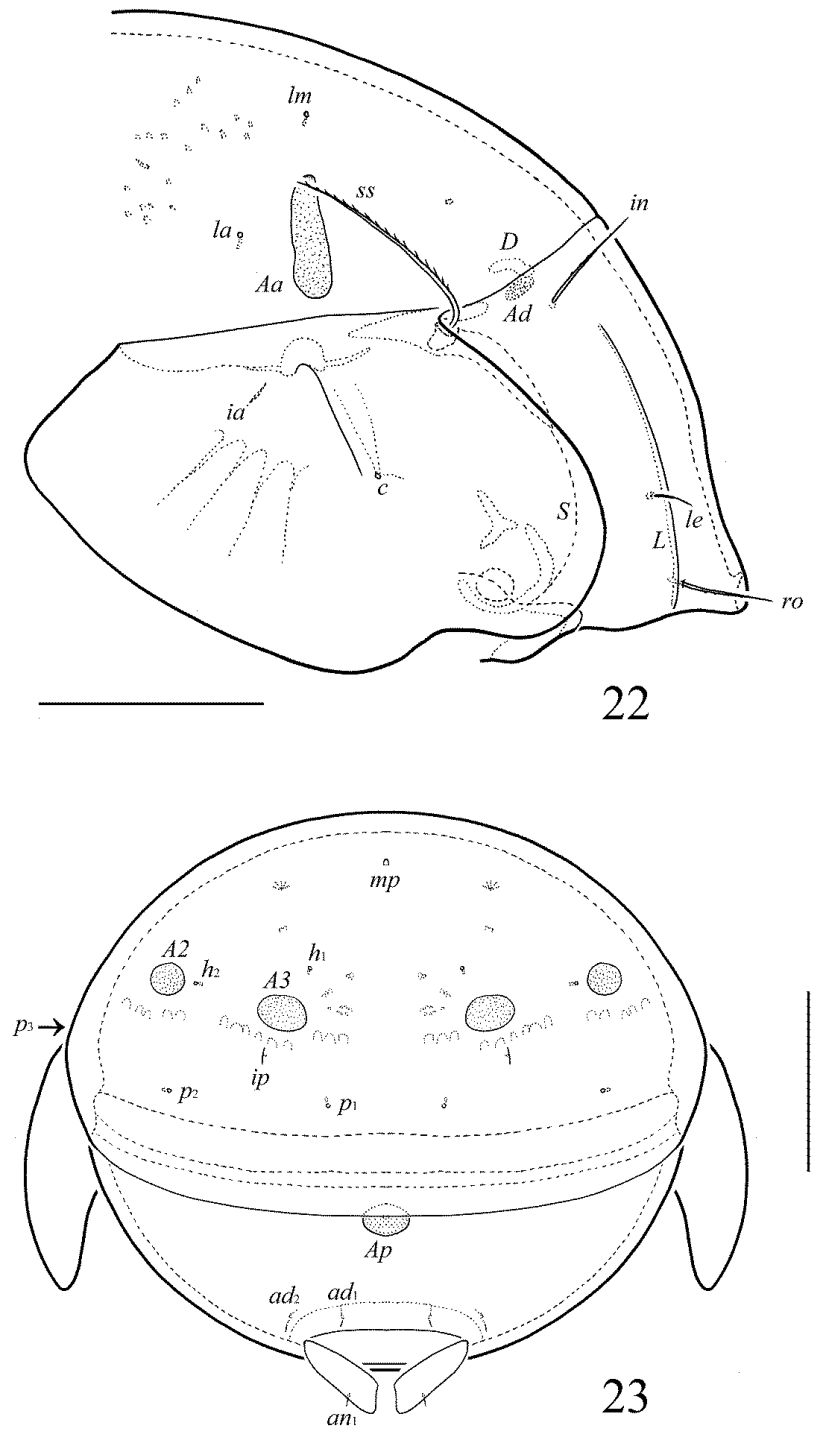
Galumna (Galumna) indonesica sp. n., adult: **22** anterior part of body, lateral view (gnathosoma and leg I not shown) **23** posterior view. Scale bars 100 µm.

**Figures 24–28.**
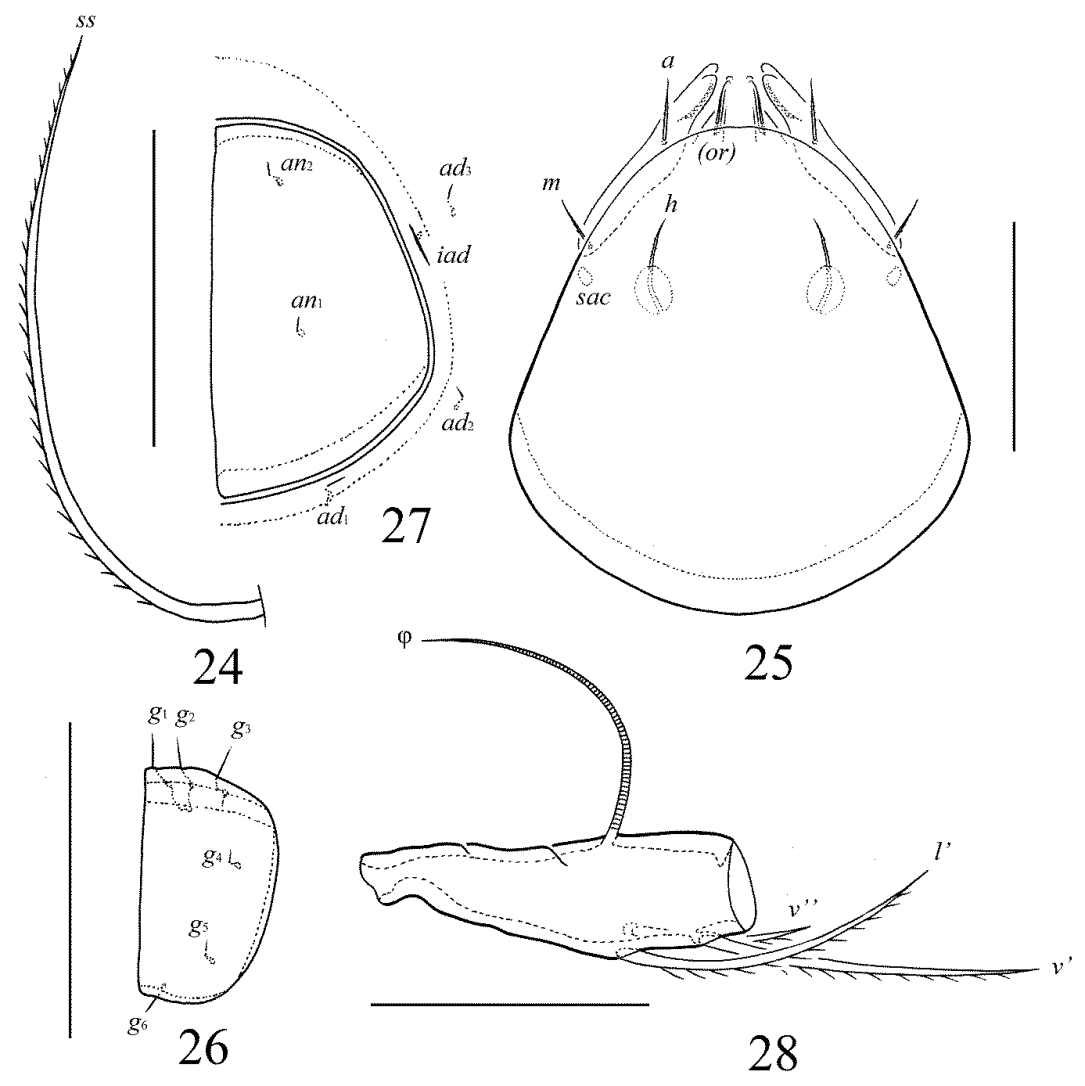
Galumna (Galumna) indonesica sp. n., adult: **24** bothridial seta **25** subcapitulum, ventral view **26** genital plate, left **27** anal plate, left, and adanal setae **28** tibia of leg IV, left, antiaxial view. Scale bars 50 µm.

#### Material examined.

Holotype (female) and three paratypes (two females and one male): Indonesia, Sumatra, Bukit Duabelas landscape, jungle rubber agroforest, research site BJ5, 02°08'35.6"S, 102°51'04.7"E, 51 m a.s.l., in upper soil layer (0–5 cm). All specimens were collected by Bernhard Klarner (Nov. 2013) and identified and collected to morphospecies level by Dorothee Sandmann.

#### Type deposition.

The holotype is deposited in LIPI (Indonesian Institute of Science) Cibinong, Indonesia; two paratypes are deposited in the collection of the Senckenberg Museum, Görlitz, Germany; one paratype is deposited in the collection of the Tyumen State University Museum of Zoology, Tyumen, Russia.

#### Etymology.

The specific name *indonesica* refers to the country of origin, Indonesia.

#### Remarks.

Galumna (Galumna) indonesica sp. n. is morphologically most similar to Galumna (Galumna) parakazakhstani Ermilov & Anichkin, 2014 from Vietnam (see Ermilov and Anichkin 2014a) in having lamellar lines directed to the anterior part of the prodorsum, setiform and ciliate bothridial setae, four pairs of notogastral porose areas with *Aa* elongated and transversally oriented, a median pore and an elongated postanal porose area. However, the new species differs from the latter by the position of rostral setae (nearly to the lamellar lines vs. distanced in Galumna (Galumna) parakazakhstani), the length of rostral and lamellar setae (rostral setae longer vs. lamellar setae longer in Galumna (Galumna) parakazakhstani) and the presence of anterior notogastral margin (vs. absent in Galumna (Galumna) parakazakhstani).

### Galumna (Galumna) mikoi sp. n.

Taxon classificationAnimaliaOribatidaGalumnidae

http://zoobank.org/ABED7400-EDB6-4D8F-9667-AD3426677A24

Figs 29 , 30 , 31–32 , 33–37

#### Diagnosis.

Body size: 258–287 × 184–204. Surface of anogenital region and medio-anterior part of notogaster foveolate, surface of subcapitular mentum, genital and anal plates, antero-lateral parts of pteromorphs and posterior part of notogaster striate. Rostral and lamellar setae of medium size, interlamellar setae minute. Bothridial setae clavate. Anterior notogastral margin developed. Four pairs of rounded porose areas present on notogaster. Median pore and postanal porose area present.

#### Description.

*Measurements*. Body length: 258 (holotype: male), 258–287 (three paratypes: two females and one male); notogaster width: 188 (holotype), 184–204 (three paratypes). Without sexual dimorphism.

*Integument*. Body color brown. Surface of anogenital region and medio-anterior part of notogaster foveolate (diameter of foveolae up to 6). Surface of subcapitular mentum, genital and anal plates, antero-lateral parts of pteromorphs and posterior part of notogaster striate.

*Prodorsum*. Rostrum rounded. Lamellar and sublamellar lines parallel, curving backwards. Rostral and lamellar setae similar in length (20–24), setiform, slightly barbed. Interlamellar setae minute (1). Bothridial setae (49–57) clavate, with long, smooth stalk and rounded, barbed head. Exobothridial setae and their alveoli absent. Porose areas *Ad* oval, transversally oriented (6 × 4).

*Notogaster*. Anterior notogastral margin developed. Dorsophragmata elongated longitudinally. Four pairs of porose areas rounded, with distinct margins: *Aa* (8–12) larger than *A1*, *A2* and *A3* (6–8). Notogastral setae represented by 10 pairs of alveoli, *la* inserted posteriorly to *Aa*. Median pore present in all specimens, located between *A2*. All lyrifissures distinct, *im* located between *lm* and *A1*. Opisthonotal gland openings located laterally to *A1*.

*Gnathosoma*. Morphology of subcapitulum, palps and chelicerae typical for Galumna (Galumna) (see Engelbrecht 1969; Ermilov and Anichkin 2010). Subcapitulum size: 73–77 × 65–69. Subcapitular setae *a* (12–14) setiform, slightly barbed, longer and thicker than minute, smooth *m* and *h* (both pairs 4). Two pairs of adoral setae (8) setiform, hook-like distally, barbed. Palps (57) with typical setation: 0–2–1–3–9(+ω). Axillary sacculi distinct. Chelicerae (90) with two setiform, barbed setae; *cha* (32) longer than *chb* (20). Trägårdh’s organ long, tapered.

*Epimeral and lateral podosomal regions*. Anterior tectum of epimere I smooth. Apodemes 1, 2, sejugal and 3 well visible. Setal formula: 1–0–1–1. Setae thin, smooth, *3b* (8–10) longer than *1a* and *4a* (4). Pedotecta II rounded anteriorly in ventral view. Discidia triangular. Circumpedal carinae distinct, clearly not reach the insertions of setae *3b*.

*Anogenital region*. Six pairs of genital (*g*_1_, 8; *g*_2_, 6; *g*_3_–*g*_6_, 4), one pair of aggenital (4), two pairs of anal (4) and three pairs of adanal (4) setae thin, smooth. Three setae on anterior edge of each genital plate. Adanal setae distanced equal from each other, inserted in one diagonal row on each side of adanal region. Adanal setae *ad*_3 _inserted postero-laterally to adanal lyrifissures. Postanal porose area oval, transversally oriented (6–10 × 4–6).

*Legs*. Morphology of leg segments, setae and solenidia typical for Galumna (Galumna) (see Engelbrecht 1969; Ermilov and Anichkin 2010). Tridactylous, claws smooth. Formulas of leg setation and solenidia are similar to Galumna (Atypicogalumna) corpuzrarosae sp. n. (Table 1). Solenidion φ of tibiae IV inserted dorsally at about 2/3 length of segment.

**Figure 29.**
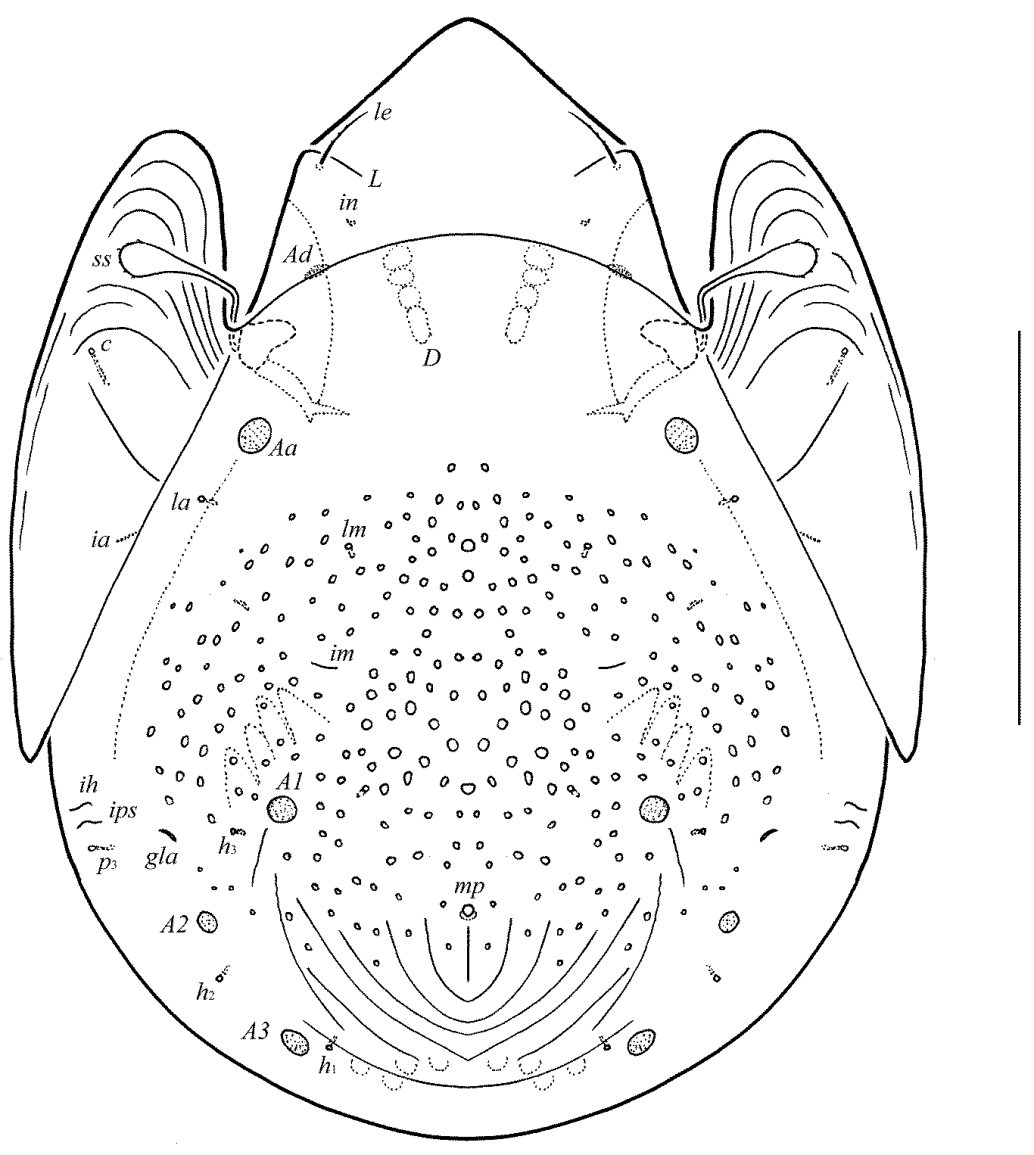
Galumna (Galumna) mikoi sp. n., adult: dorsal view. Scale bar 100 µm.

**Figure 30.**
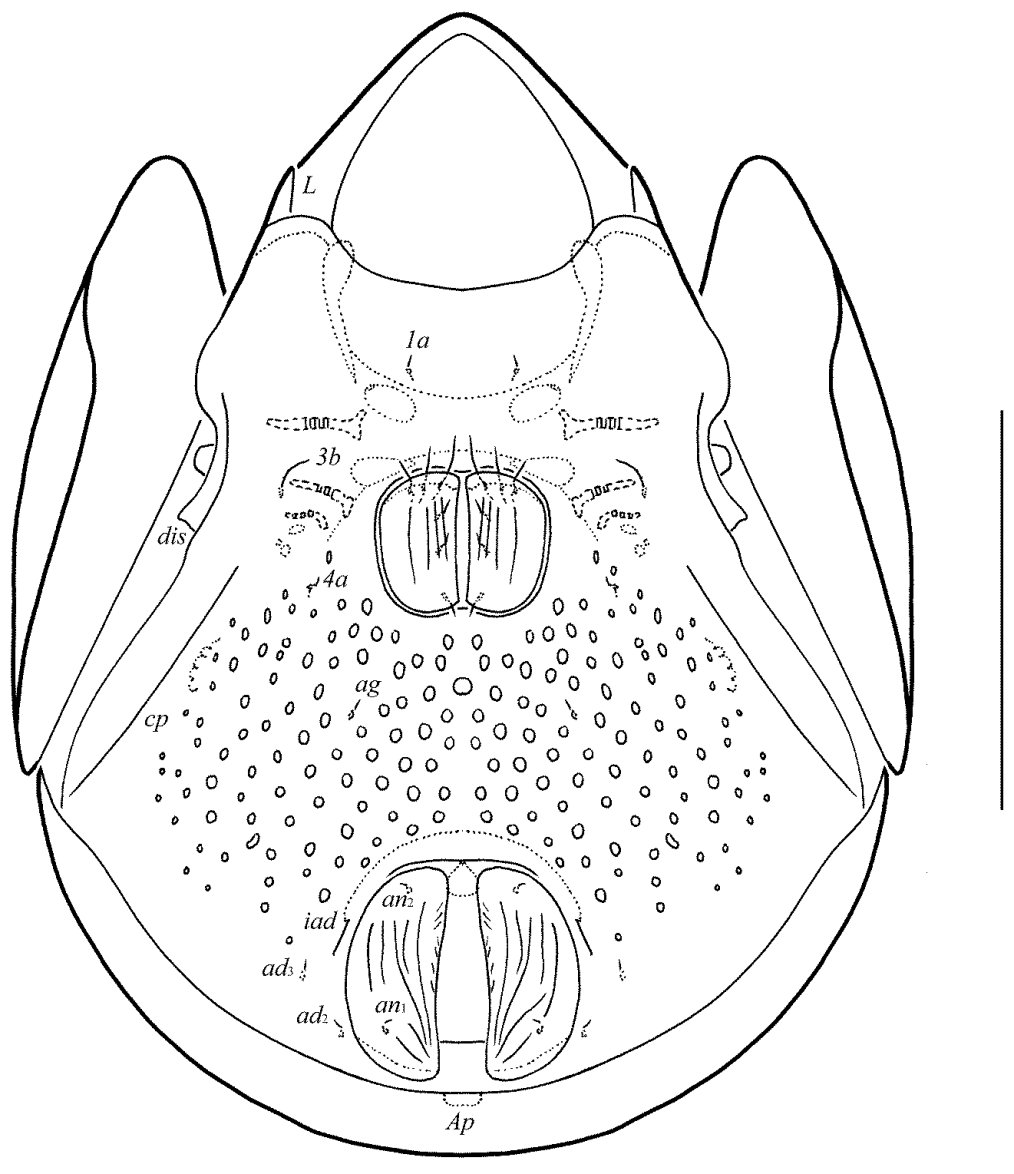
Galumna (Galumna) mikoi sp. n., adult: ventral view (gnathosoma and legs not shown). Scale bar 100 µm.

**Figures 31–32.**
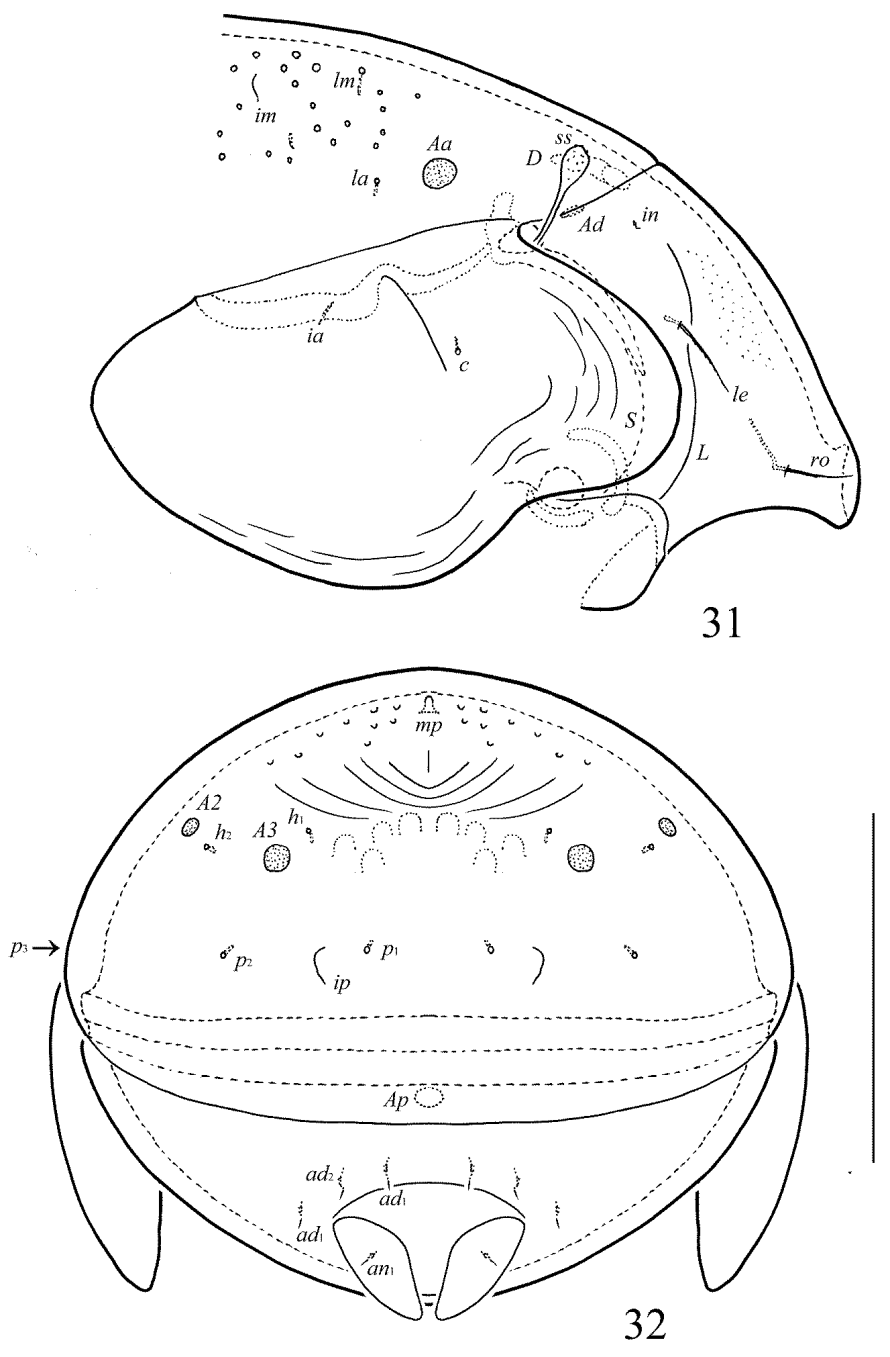
Galumna (Galumna) mikoi sp. n., adult: **31** anterior part of body, lateral view (gnathosoma and leg I not shown) **32** posterior view. Scale bar 100 µm.

**Figures 33–37.**
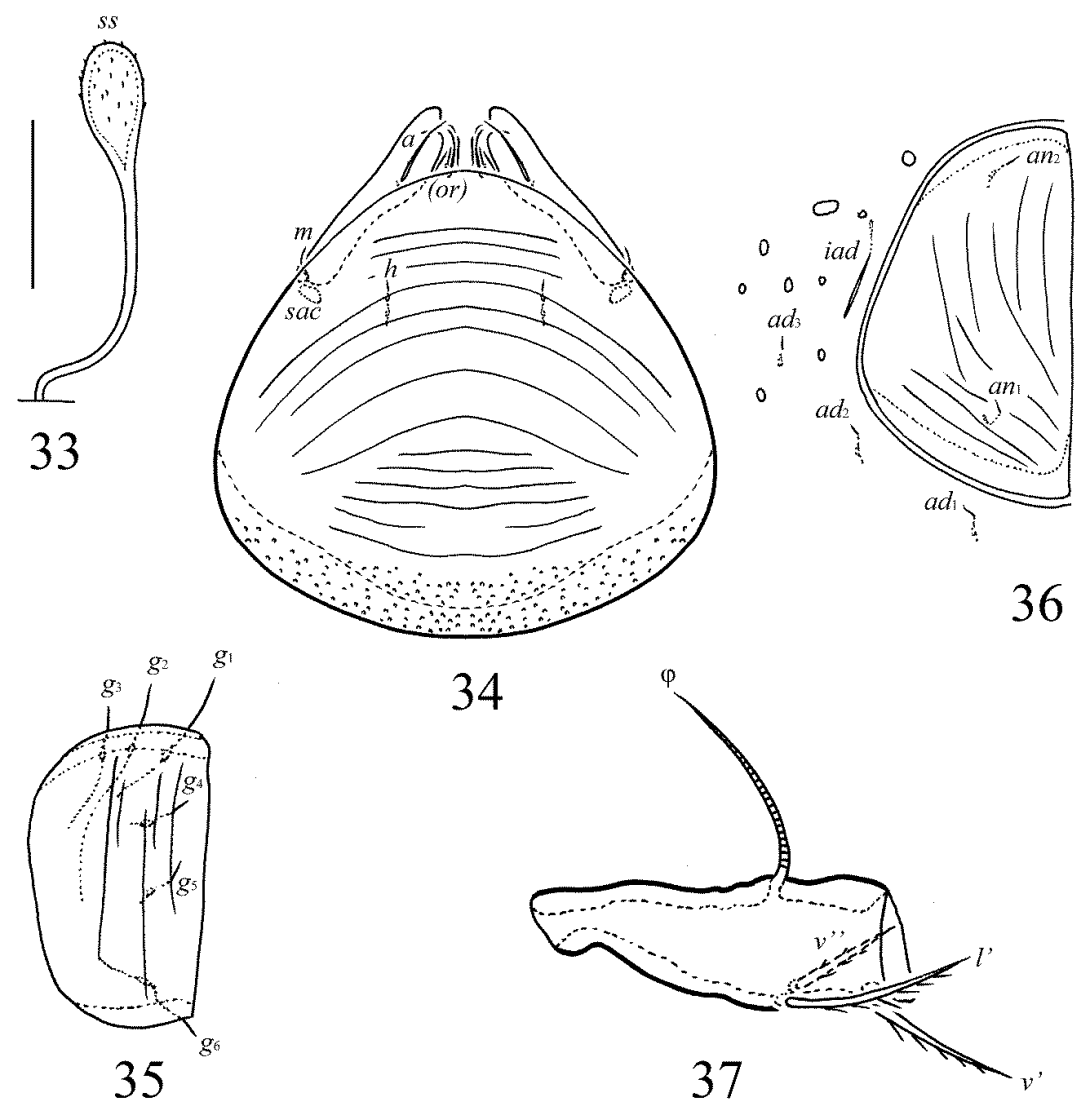
Galumna (Galumna) mikoi sp. n., adult: **33** bothridial seta **34** subcapitulum, ventral view **35** genital plate, right **36** anal plate, right, and adanal setae **37** tibia of leg IV, left, antiaxial view. Scale bar 20 µm.

#### Material examined.

Holotype (male): Indonesia, Sumatra, Harapan landscape, jungle rubber agroforest, research site HJ4, 01°47'07.3"S, 103°16'36.9"E, 57 m a.s.l., in forest floor litter material. Three paratypes (two females and one male): Indonesia, Sumatra, Harapan landscape, secondary rainforest, research site HF4, 02°11'15.2"S, 103°20'33.4"E, 77 m a.s.l., in forest floor litter material. All specimens were collected by Bernhard Klarner (Nov. 2013) and identified and collected to morphospecies level by Dorothee Sandmann.

#### Type deposition.

The holotype is deposited in LIPI (Indonesian Institute of Science) Cibinong, Indonesia; two paratypes are deposited in the collection of the Senckenberg Museum, Görlitz, Germany; one paratype is deposited in the collection of the Tyumen State University Museum of Zoology, Tyumen, Russia.

#### Etymology.

The specific name is dedicated to our friend and colleague, acarologist, Dr. Ladislav Miko (Czech University of Life Sciences Prague, Charles University in Prague, Prague, Czech Republic).

#### Remarks.

Galumna (Galumna) mikoi sp. n. is morphologically most similar to Galumna (Galumna) innexa Pérez-Íñigo & Baggio, 1986 from the Neotropical region (see Pérez-Íñigo and Baggio 1986) in having striate pteromorphs, minute interlamellar setae, anterior notogastral margin, four pairs of rounded porose areas on notogaster and median pore. However, the new species differs from the latter by the foveolate notogaster and anogenital region (vs. not foveolate in Galumna (Galumna) innexa), striate posterior part of notogaster (vs. not striate in Galumna (Galumna) innexa) and clavate bothridial setae (vs. lanceolate in Galumna (Galumna) innexa).

### Galumna (Cosmogalumna) areticulata sp. n.

Taxon classificationAnimaliaOribatidaGalumnidae

http://zoobank.org/3177798F-4D88-4538-9862-C51DD5495C85

Figs 38 , 39 , 40–41 , 42–46

#### Diagnosis.

Body size: 298–315 × 215–249. Transverse band of strong, branched cerotegumental ridges developed in middle part of notogaster and between genital and anal plates, not forming a reticulate pattern, only a few cells present exeptionally. Rostral and lamellar setae short, interlamellar setae represented by alveoli. Bothridial setae clavate. Four pairs of rounded porose areas present on notogaster. Median pore and postanal porose area present.

#### Description.

*Measurements*. Body length: 315 (holotype: male), 298–315 (seven paratypes: two females and five males); notogaster width: 249 (holotype), 215–249 (seven paratypes). Without sexual dimorphism.

*Integument*. Body color brown. Body surface, pteromorphs, genital and anal plates punctate (visible in dissected specimens). Subcapitular mentum smooth. Transverse band of strong, branched cerotegumental ridges developed in the middle part of the notogaster and between the genital and anal plates. These ridges comparatively short and not forming a clear reticulate pattern, only a few cells present exeptionally.

*Prodorsum*. Rostrum rounded. Lamellar and sublamellar lines parallel, curving backwards. Rostral setae (18–20) thin, smooth, pressed to the surface of prodorsum. Lamellar setae (6–8) minute. Interlamellar setae represented by alveoli. Bothridial setae (53–57) clavate, with long stalk and short head, rounded and smooth to slightly roughened distally. Exobothridial setae and their alveoli absent. Porose areas *Ad* oval, transversally oriented (6–8 × 4–6).

*Notogaster*. Anterior notogastral margin developed. Dorsophragmata elongated longitudinally. Four pairs of porose areas rounded, with distinct margins: *Aa* (12–16) slightly larger than *A1*, *A2* and *A3* (all 8–10). Notogastral setae represented by 10 pairs of alveoli, *la* inserted posteriorly to *Aa*. Median pore present in all specimens, located between *A2*. All lyrifissures distinct, *im* located between *lm* and *lp.* Opisthonotal gland openings located laterally to *A1*.

*Gnathosoma*. Morphology of subcapitulum, palps and chelicerae typical for Galumna (Cosmogalumna) (see Ermilov et al. 2011; Ermilov and Anichkin 2013). Subcapitulum size: 77–82 × 61–65. Subcapitular setae setiform, indistinctly barbed, *h* (4) shorter than *m* (6) and *a* (10–12), *a* thickest, *h* thinnest. Two pairs of adoral setae (6) setiform, hook-like distally, indistinctly barbed. Palps (69) with typical setation: 0–2–1–3–9(+ω). Axillary sacculi distinct. Chelicerae (94) with two setiform, barbed setae; *cha* (32) longer than *chb* (20). Trägårdh’s organ long, tapered.

*Epimeral and lateral podosomal regions*. Anterior tectum of epimere I smooth. Apodemes 1, 2, sejugal and 3 well visible. Setal formula: 1–0–1–2. Setae thin, smooth, *3b* (6) slightly longer than *1a*, *4a* and *4b* (4). Pedotecta II roundly triangular in ventral view. Discidia triangular. Circumpedal carinae distinct, clearly not reaching the insertions of setae *3b*.

*Anogenital region*. Six pairs of genital (*g*_1_, *g*_2_, 6–8; *g*_3_–*g*_6_, 4), one pair of aggenital (4), two pairs of anal (4) and three pairs of adanal (4) setae thin, smooth. Three setae on anterior edge of each genital plate. Adanal setae *ad*_3 _inserted antero-laterally to adanal lyrifissures. Postanal porose area oval, transversally oriented (8–12 × 4).

*Legs*. Morphology of leg segments, setae and solenidia typical for Galumna (Cosmogalumna) (see Ermilov et al. 2011; Ermilov and Anichkin 2013). Tridactylous, claws smooth. Formulas of leg setation and solenidia are similar to Galumna (Atypicogalumna) corpuzrarosae sp. n. (Table 1). Solenidion φ of tibiae IV inserted dorsally at about 2/3 length of segment.

**Figure 38.**
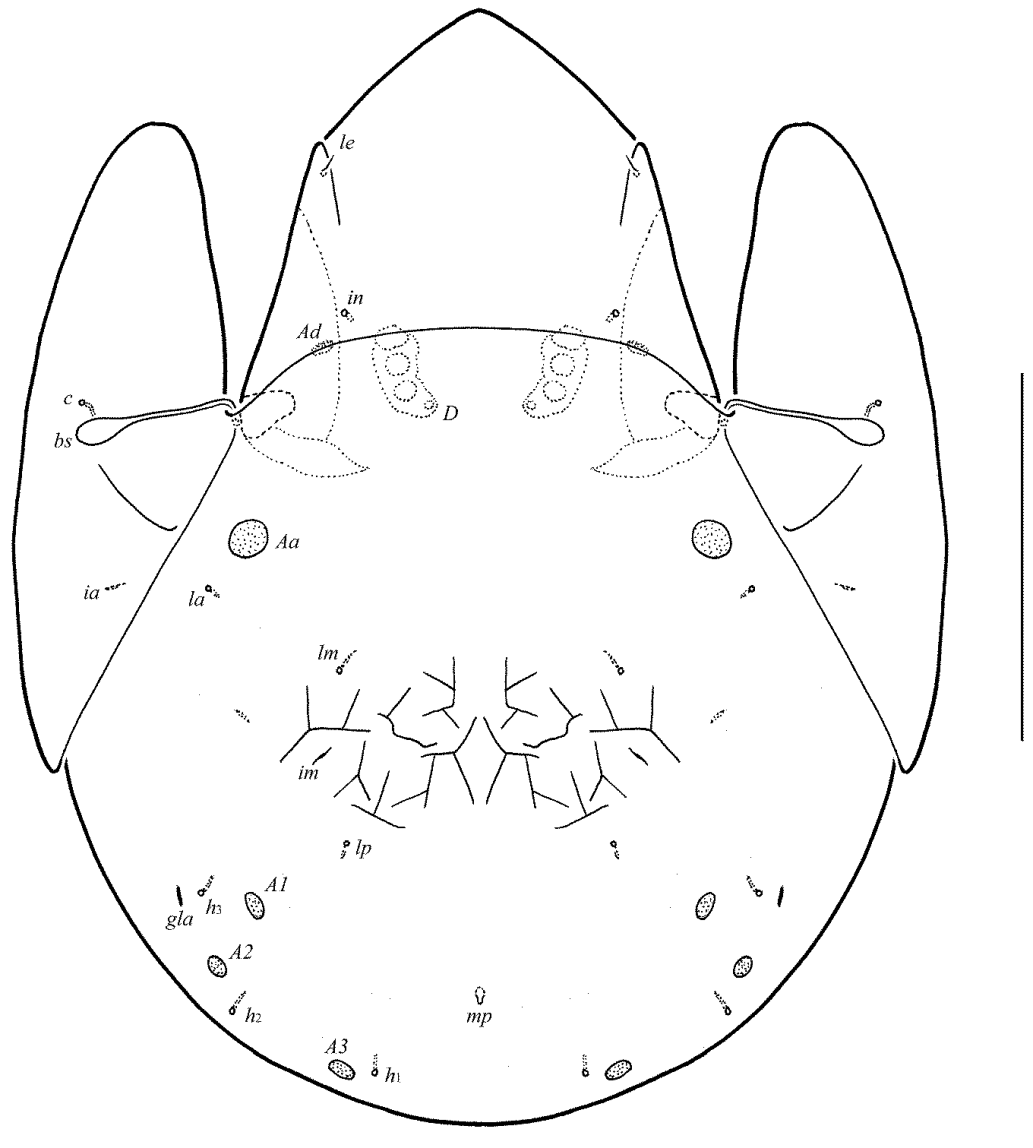
Galumna (Cosmogalumna) areticulata sp. n., adult: dorsal view. Scale bar 100 µm.

**Figure 39.**
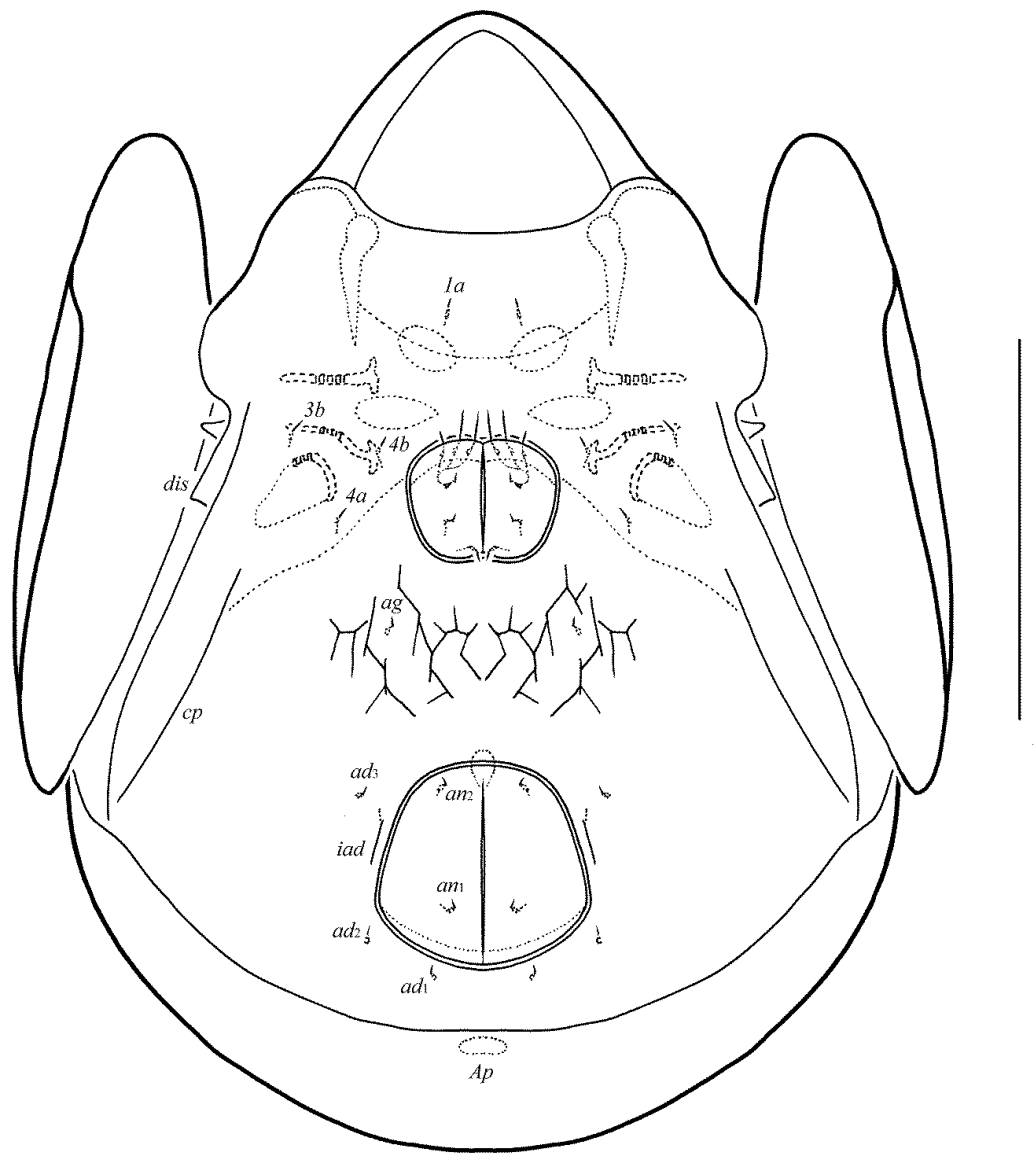
Galumna (Cosmogalumna) areticulata sp. n., adult: ventral view (gnathosoma and legs not shown). Scale bar 100 µm.

**Figures 40–41.**
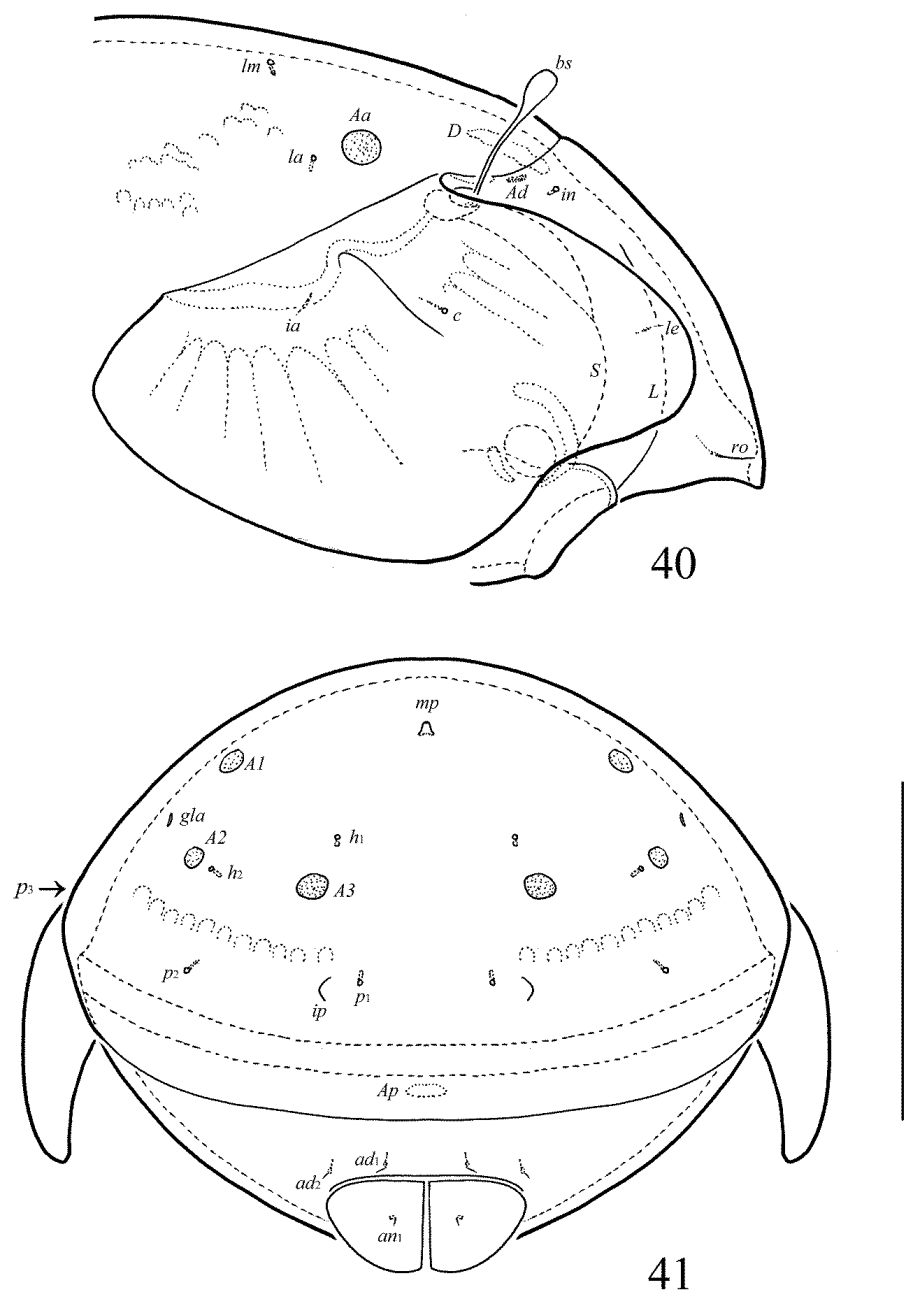
Galumna (Cosmogalumna) areticulata sp. n., adult: **40** anterior part of body, lateral view (gnathosoma and leg I not shown) **41** posterior view. Scale bar 100 µm.

**Figures 42–46.**
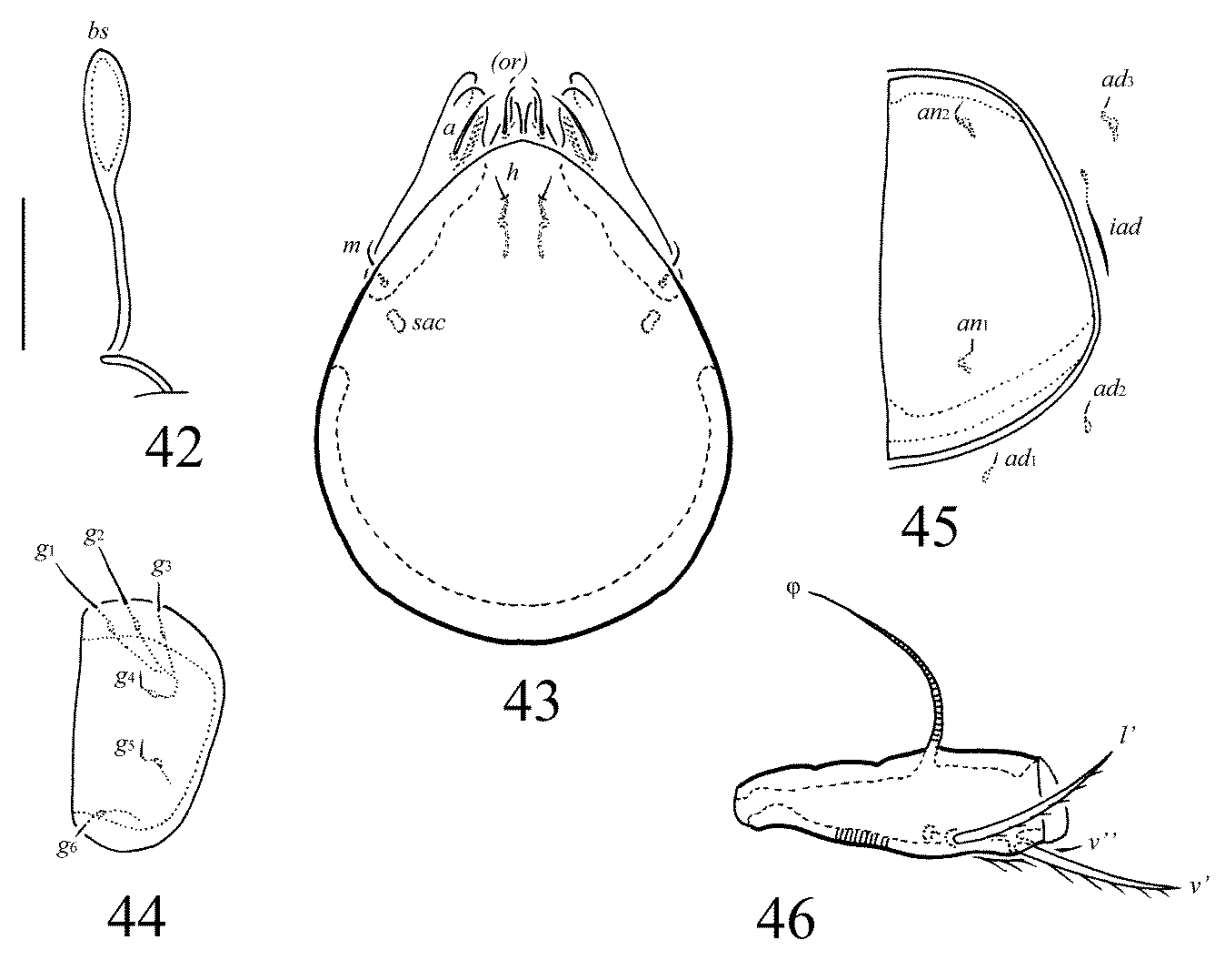
Galumna (Cosmogalumna) areticulata sp. n., adult: **42** bothridial seta **43** subcapitulum, ventral view **44** genital plate, left **45** anal plate, left, and adanal setae **46** tibia of leg IV, left, antiaxial view. Scale bar 20 µm.

#### Material examined.

Holotype (male) and two paratypes (one female and one male): Indonesia, Sumatra, Bukit Duabelas landscape, secondary rainforest, research site BF2, 01° 58'55.1"S, 102°45'02.7"E, 77 m a.s.l., in upper soil layer (0–5 cm). Five paratypes (one female and four males): Indonesia, Sumatra, Harapan landscape, jungle rubber agroforest, research site HJ1, 01°55'40.0"S, 103°15'33.8"E, 51 m a.s.l., in forest floor litter material. All specimens were collected by Bernhard Klarner (Nov. 2013) and identified and collected to morphospecies level by Dorothee Sandmann.

#### Type deposition.

The holotype is deposited in LIPI (Indonesian Institute of Science) Cibinong, Indonesia; two paratypes are deposited in the collection of the Senckenberg Museum, Görlitz, Germany; six paratypes are deposited in the collection of the Tyumen State University Museum of Zoology, Tyumen, Russia.

#### Etymology.

The specific name *areticulata* refers to the absence of clear reticulate pattern on the body.

#### Remarks.

Galumna (Cosmogalumna) areticulata sp. n. is morphologically most similar to Galumna (Cosmogalumna) praeoccupata Subías, 2004 from southern China and Vietnam (see Aoki and Hu 1993; including personal data based on the Vietnamese specimens) in having transverse band of reticulation in the middle part of the notogaster and between genital and anal plates, and the absence of striate and reticulate pattern on the prodorsum and pteromorphs. However, the new species differs from the latter by the presence of strong, branched cerotegumental ridges, which do not form a reticulate pattern (vs. distinct reticulate pattern, represented by small, numerous, dense cells in Galumna (Cosmogalumna) praeoccupata), minute lamellar setae (vs. well developed in Galumna (Cosmogalumna) praeoccupata) and the directions of lamellar lines (to anterior tectum of ventral plate vs. to acetabula I in Galumna (Cosmogalumna) praeoccupata).

### Galumna (Cosmogalumna) sumatrensis sp. n.

Taxon classificationAnimaliaOribatidaGalumnidae

http://zoobank.org/D757C52A-EB98-4DE5-9D13-D3196FF12A7F

Figs 47 , 48 , 49–50 , 51–55

#### Diagnosis.

Body size: 282–298 × 182–215. Reticulate pattern in the middle part of notogaster represented by few large cells, reticulate pattern between genital and anal plates represented by small, numerous, dense cells. Rostral and lamellar setae thin, indistinctly barbed, interlamellar setae represented by alveoli. Bothridial setae clavate. Four pairs of rounded porose areas on notogaster. Median pore absent. Postanal porose area present.

#### Description.

*Measurements*. Body length: 282 (holotype: male), 282, 298 (two paratypes: one female and one male); notogaster width: 215 (holotype), 182, 215 (two paratypes). Without sexual dimorphism.

*Integument*. Body color brown. Body surface, pteromorphs, genital and anal plates, and subcapitular mentum punctate. Reticulate pattern in the middle part of notogaster present, cells large and not numerous. Reticulate pattern between genital and anal plates represented by small, numerous, dense cells.

*Prodorsum*. Rostrum rounded. Lamellar and sublamellar lines parallel, curving backwards. Rostral (16) and lamellar (10–12) setae thin, indistinctly barbed. Interlamellar setae represented by alveoli. Bothridial setae (49–53) clavate, with long stalk and short head, rounded and barbed distally. Exobothridial setae and their alveoli absent. Porose areas *Ad* oval, transversally oriented (14–16 × 4–6).

*Notogaster*. Anterior notogastral margin developed. Dorsophragmata large, elongated longitudinally. Four pairs of porose areas rounded, with distinct margins: *Aa* (14–16) larger than *A1*, *A2* and *A3* (all 8–10). Notogastral setae represented by 10 pairs of alveoli, *la* inserted posteriorly to *Aa*. Median pore absent in all specimens. All lyrifissures distinct, *im* located between *lm* and *A1*. Opisthonotal gland openings located antero-laterally to *A2*.

*Gnathosoma*. Morphology of subcapitulum, palps and chelicerae typical for Galumna (Cosmogalumna) (see Ermilov et al. 2011; Ermilov and Anichkin 2013). Subcapitulum size: 77 × 65–69. Subcapitular setae setiform, indistinctly barbed, *h* and *m* (all 6) shorter than *a* (12–14), *a* thickest, *h* thinnest. Two pairs of adoral setae (8) setiform, hook-like distally, indistinctly barbed. Palps (69) with typical setation: 0–2–1–3–9(+ω). Axillary sacculi distinct. Chelicerae (94) with two setiform, barbed setae; *cha* (32) longer than *chb* (20). Trägårdh’s organ long, tapered.

*Epimeral and lateral podosomal regions*. Anterior tectum of epimere I smooth. Apodemes 1, 2, sejugal and 3 well visible. Setal formula: 1–0–1–1. Setae *1a*, *3b* and *4a* similar in length (4), thin, smooth. Pedotecta II roundly triangular in ventral view. Discidia triangular. Circumpedal carinae distinct, clearly not reaching the insertions of setae *3b*.

*Anogenital region*. Six pairs of genital (*g*_1_, *g*_2_, 8; *g*_3_–*g*_6_, 4), one pair of aggenital (4), two pairs of anal (4) and three pairs of adanal (4) setae thin, smooth. Three setae on anterior edge of each genital plate. Adanal setae *ad*_3 _inserted laterally to adanal lyrifissures. Postanal porose area oval, transversally oriented (12–20 × 4–8).

*Legs*. Morphology of leg segments, setae and solenidia typical for Galumna (Cosmogalumna) (see Ermilov et al. 2011; Ermilov and Anichkin 2013). Tridactylous, claws smooth. Formulas of leg setation and solenidia are similar to Galumna (Atypicogalumna) corpuzrarosae sp. n. (Table 1). Solenidion φ of tibiae IV inserted dorsally at about 2/3 length of segment.

**Figure 47.**
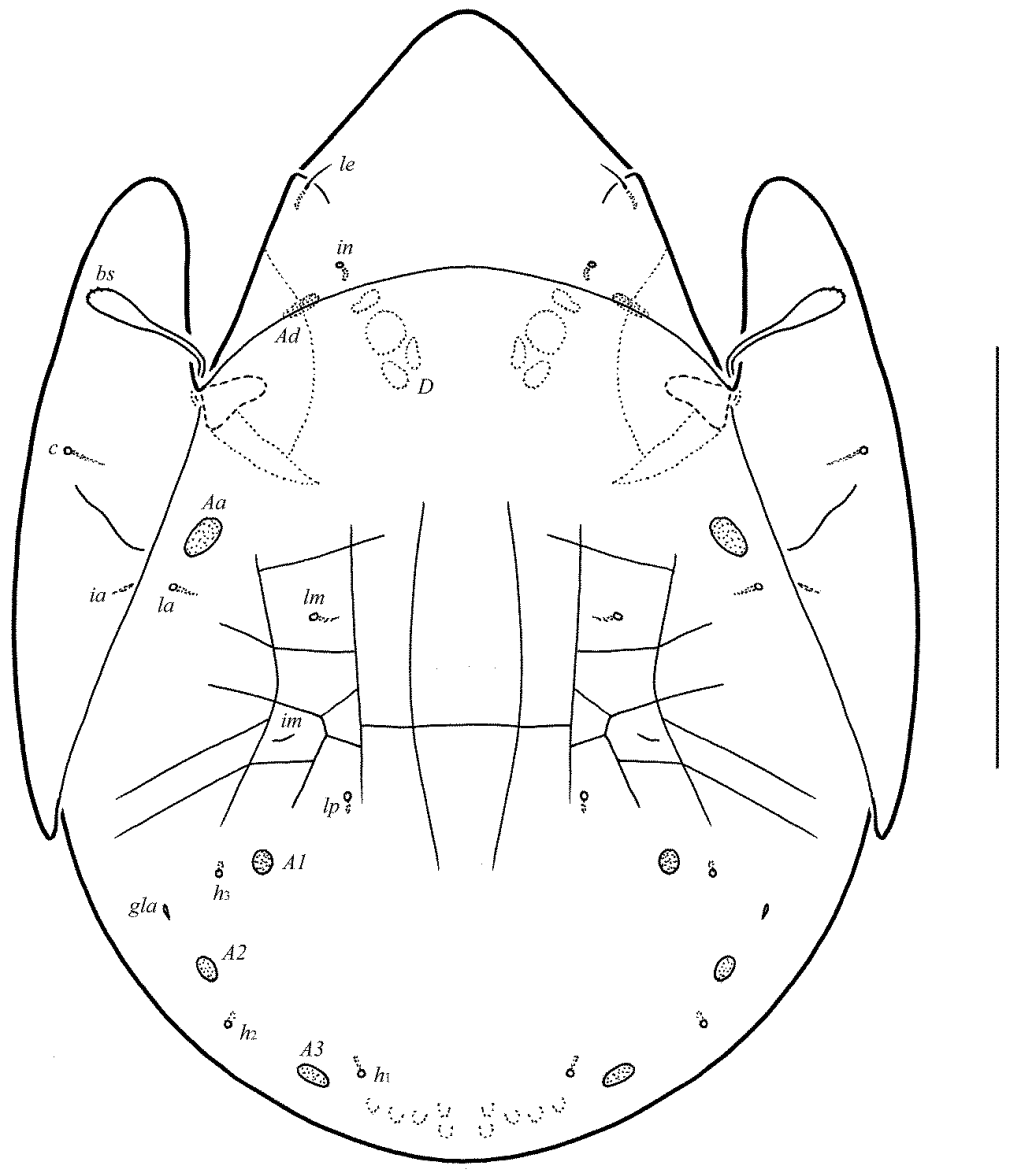
Galumna (Cosmogalumna) sumatrensis sp. n., adult: dorsal view. Scale bar 100 µm.

**Figure 48.**
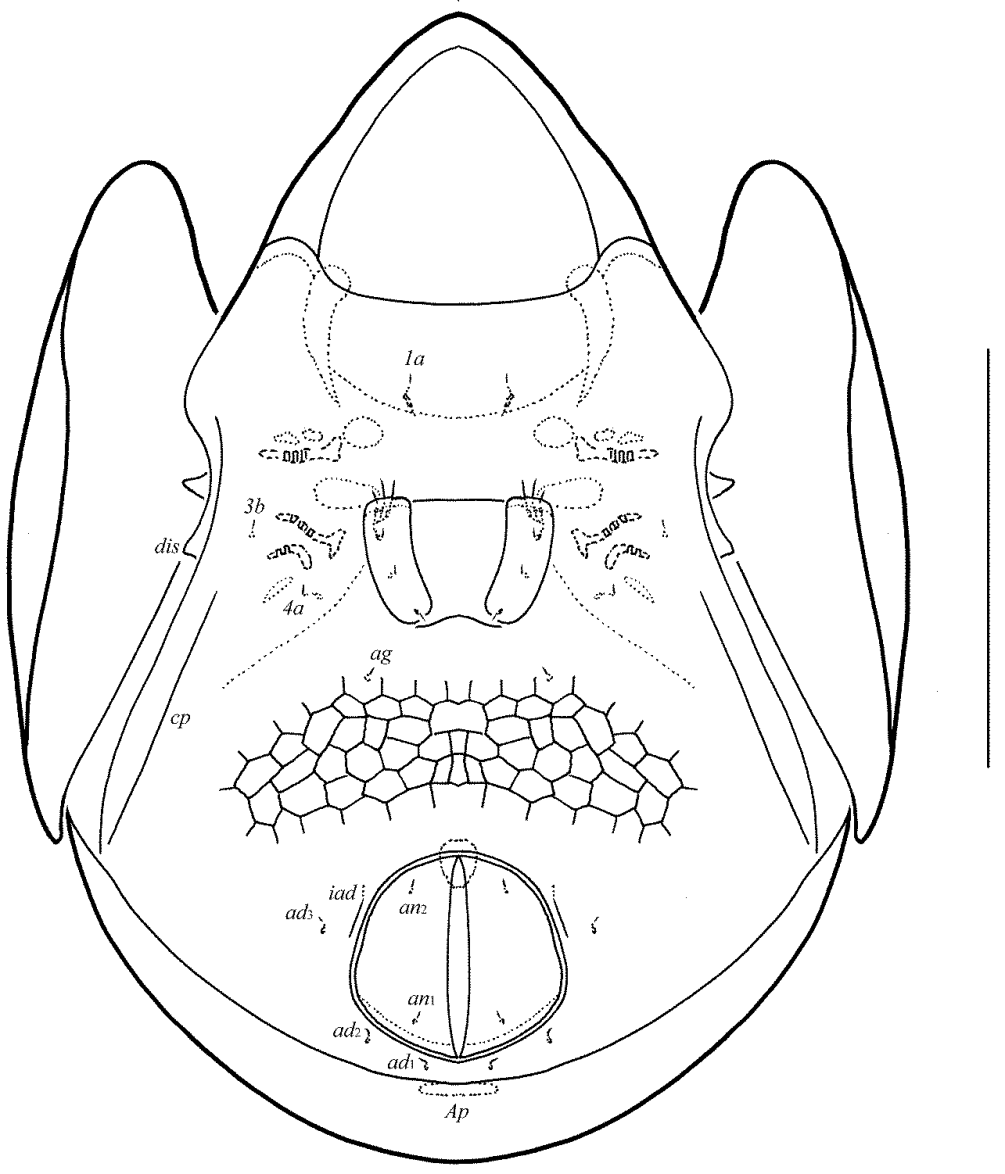
Galumna (Cosmogalumna) sumatrensis sp. n., adult: ventral view (gnathosoma and legs not shown). Scale bar 100 µm.

**Figures 49–50.**
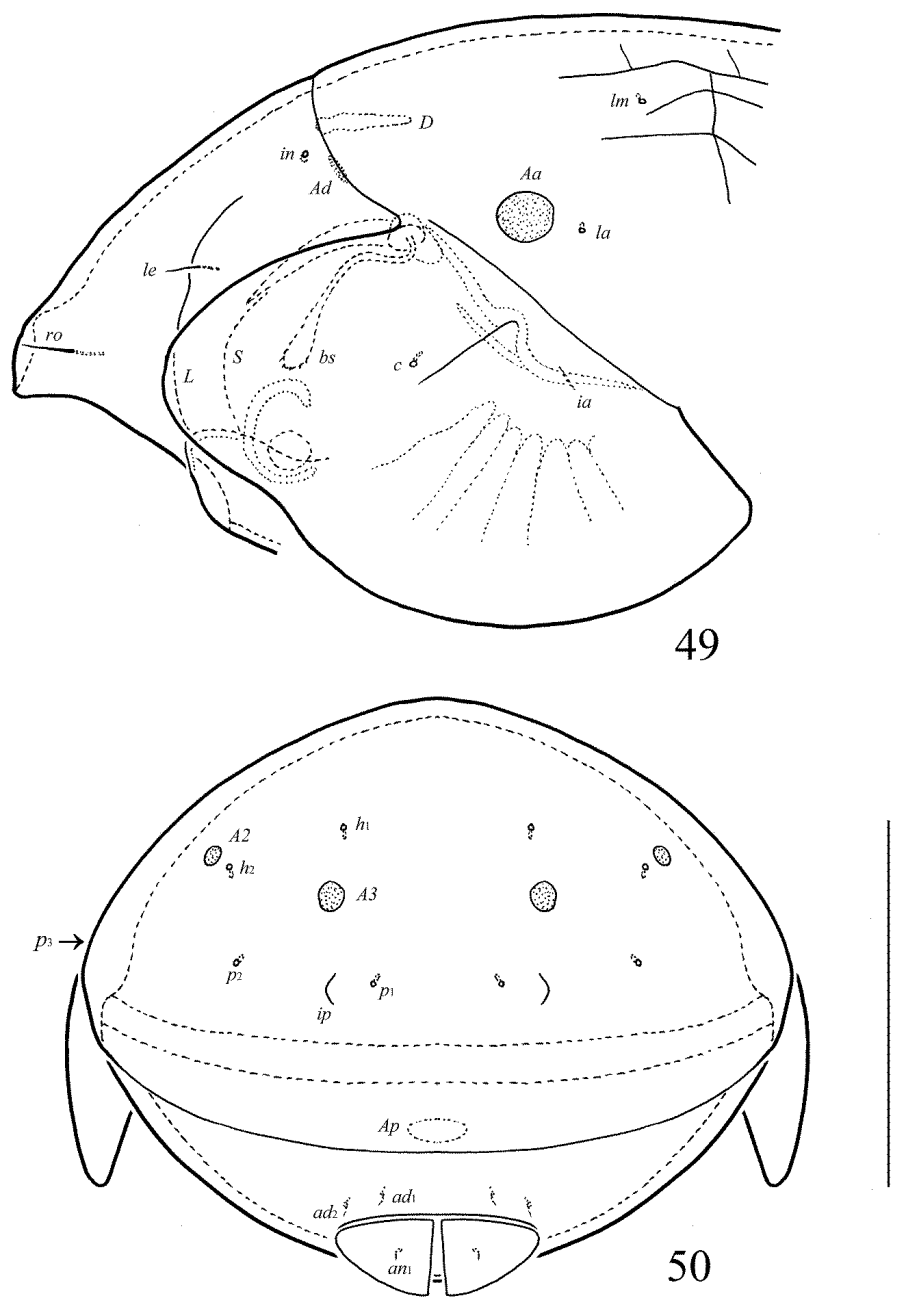
Galumna (Cosmogalumna) sumatrensis sp. n., adult: **49** anterior part of body, lateral view (gnathosoma and leg I not shown) **50** posterior view. Scale bar 100 µm.

**Figures 51–55.**
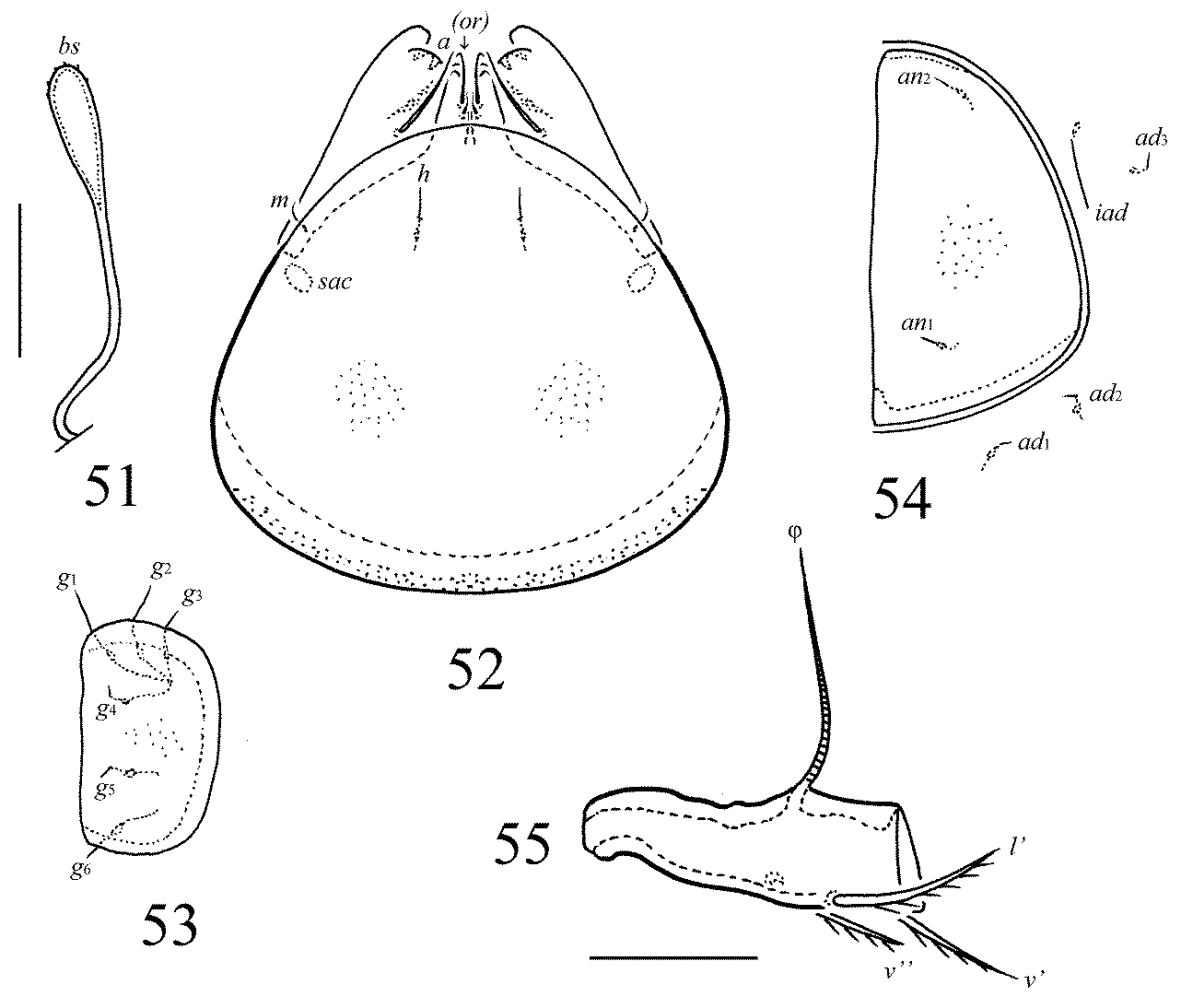
Galumna (Cosmogalumna) sumatrensis sp. n., adult: **51** bothridial seta **52** subcapitulum, ventral view **53** genital plate, left **54** anal plate, left, and adanal setae **55** tibia of leg IV, left, antiaxial view. Scale bars 20 µm.

#### Material examined.

Holotype (male): Indonesia, Sumatra, Harapan landscape, secondary rainforest, research site HF4, 02°11'15.2"S, 103°20'33.4"E, 77 m a.s.l., in forest floor litter material. Two paratypes (one female and one male): Indonesia, Sumatra, Harapan landscape, secondary rainforest, research site HF4, 02°11'15.2"S, 103°20'33.4"E, 77 m a.s.l., in upper soil layer (0–3 cm). All specimens were collected by Bernhard Klarner (Nov. 2013) and identified and collected to morphospecies level by Dorothee Sandmann.

#### Type deposition.

The holotype is deposited in LIPI (Indonesian Institute of Science) Cibinong, Indonesia; one paratype is deposited in the collection of the Senckenberg Museum, Görlitz, Germany; one paratype is deposited in the collection of the Tyumen State University Museum of Zoology, Tyumen, Russia.

#### Etymology.

The specific name *sumatrensis* refers to the island of origin, Sumatra.

#### Remarks.

Galumna (Cosmogalumna) sumatrensis sp. n. is morphologically most similar to Galumna (Cosmogalumna) praeoccupata Subías, 2004 from southern China and Vietnam (see Aoki and Hu 1993; including personal data based on the Vietnamese specimens) and Galumna (Cosmogalumna) areticulata sp. n. from Indonesia in having a transverse band of reticulation in the middle part of the notogaster and between genital and anal plates, and the absence of striate and reticulate pattern on the prodorsum and pteromorphs. The new species differs from Galumna (Cosmogalumna) praeoccupata by the presence of large and not numerous of reticulate cells on notogaster (vs. pattern distinct, represented by small, numerous, dense cells in Galumna (Cosmogalumna) praeoccupata) and absence of median pore (vs. present in Galumna (Cosmogalumna) praeoccupata). The new species differs from Galumna (Cosmogalumna) areticulata sp. n. by the presence of reticulate pattern in the anogenital region represented by small, numerous, dense cells (vs. strong, branched cerotegumental ridges, which do not form a reticulate pattern present in Galumna (Cosmogalumna) areticulata sp. n.) and the absence a median pore (vs. present in Galumna (Cosmogalumna) areticulata sp. n.).

### Galumna (Neogalumna) specifica sp. n.

Taxon classificationAnimaliaOribatidaGalumnidae

http://zoobank.org/259FF377-30D3-4B95-8E74-12D956CC4F96

Figs 56 , 57 , 58–59 , 60–64

#### Diagnosis.

Body size: 498–531 × 348–365. Subcapitular mentum, genital plates and basal part of prodorsum striate. Lamellar lines straight, divergent to sublamellar lines medio-anteriorly. Prodorsal setae setiform, barbed, *le* thinnest. Bothridial setae, setiform, ciliate. Anterior notogastral margin developed. Notogastral setal alveoli *la* absent, *c*_x_ present. Four pairs of rounded porose areas on notogaster. Median pore absent. Postanal porose area elongate oval.

#### Description.

*Measurements*. Body length: 498 (holotype: male), 498–531 (three paratypes: one female and two males); notogaster width: 348 (holotype), 348–365 (three paratypes). Without sexual dimorphism.

*Integument*. Body color brown. Body surface, pteromorphs and anal plates smooth. Subcapitular mentum, genital plates and basal part of prodorsum with longitudinal striae.

*Prodorsum*. Rostrum rounded. Medio-anterior part slightly elongate, hump-like. Lamellar lines straight, directed to insertions of rostral setae, but clearly not reaching them. Sublamellar lines curving backwards. Rostral (39–45), lamellar (18–20) and interlamellar (18–20) setae setiform, barbed, lamellar setae thinnest. Bothridial setae long (106–114), setiform, unilaterally ciliate. Exobothridial setae and their alveoli absent. Porose areas *Ad* narrow, elongate oval, transversally oriented (28–34 × 4).

*Notogaster*. Anterior notogastral margin developed. Dorsophragmata of medium size, elongated longitudinally. Four pairs of porose areas rounded, with distinct margins: *Aa* (16–24) usually slightly larger than *A1*, *A2* and *A3* (all 12–16). Notogastral setae represented by 10 pairs of alveoli, however, based on their localization, *la* absent and *c*_x_ present. Median pore absent in all specimens. All lyrifissures distinct, *im* located between *lm* and *A1*. Opisthonotal gland openings located laterally to *A1*.

*Gnathosoma*. Morphology of subcapitulum, palps and chelicerae typical for Galumna (Neogalumna) (see Ermilov and Anichkin 2010, 2014b). Subcapitulum size: 110–114 × 98–102. Subcapitular setae setiform, indistinctly barbed, *h* (6–8) shorter than *a* and *m* (both pairs 12), *a* thickest, *h* thinnest. Two pairs of adoral setae (6–8) setiform, hook-like distally, indistinctly barbed. Palps (94) with typical setation: 0–2–1–3–9(+ω). Axillary sacculi distinct. Chelicerae (139) with two setiform, barbed setae; *cha* (45) longer than *chb* (24). Trägårdh’s organ long, tapered.

*Epimeral and lateral podosomal regions*. Anterior tectum of epimere I smooth. Apodemes 1, 2, sejugal and 3 well visible. Setal formula: 1–0–1–2. Setae thin, smooth, *3b* (32–41) longer than *1a*, *4a* and *4b* (6–8). Pedotecta II distally rounded in ventral view. Discidia triangular. Circumpedal carinae distinct, clearly not reaching the insertions of setae *3b*.

*Anogenital region*. Six pairs of genital (*g*_1_, *g*_2_, 12; *g*_3_–*g*_6_, 6–8), one pair of aggenital (6–8), two pairs of anal (12) and three pairs of adanal (12) setae thin, smooth. Two setae on anterior edge of each genital plate. Adanal setae *ad*_3 _inserted postero-medially to adanal lyrifissures. Postanal porose area elongate oval, transversally oriented (45–57 × 8–12).

*Legs*. Morphology of leg segments, setae and solenidia typical for Galumna (Neogalumna) (see Ermilov and Anichkin 2010, 2014b). Tridactylous, claws smooth. Formulas of leg setation and solenidia are similar to Galumna (Atypicogalumna) corpuzrarosae sp. n. (Table 1). Solenidion φ of tibiae IV inserted dorsally at about 2/3 length of segment.

**Figure 56.**
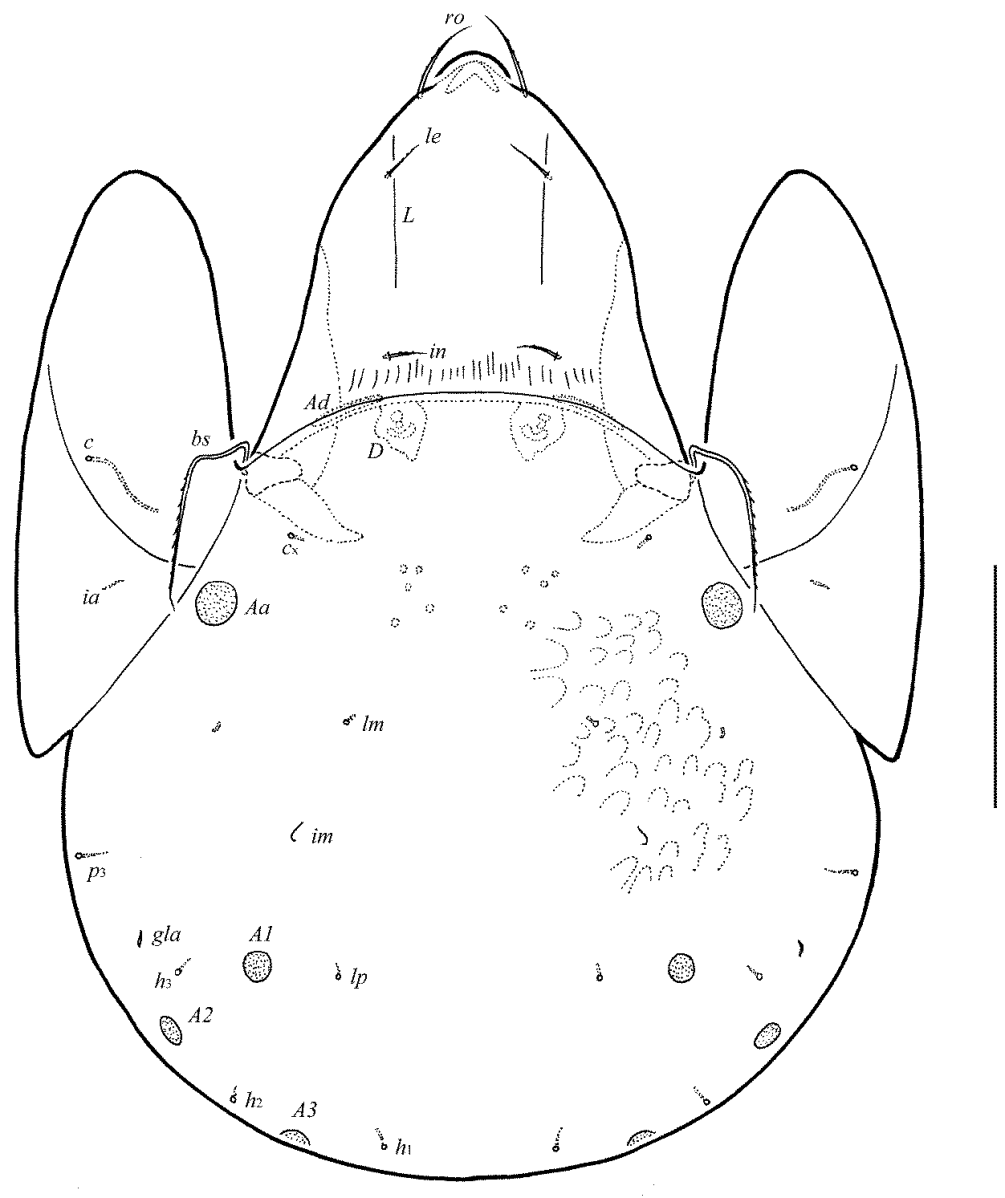
Galumna (Neogalumna) specifica sp. n., adult: dorsal view. Scale bar 100 µm.

**Figure 57.**
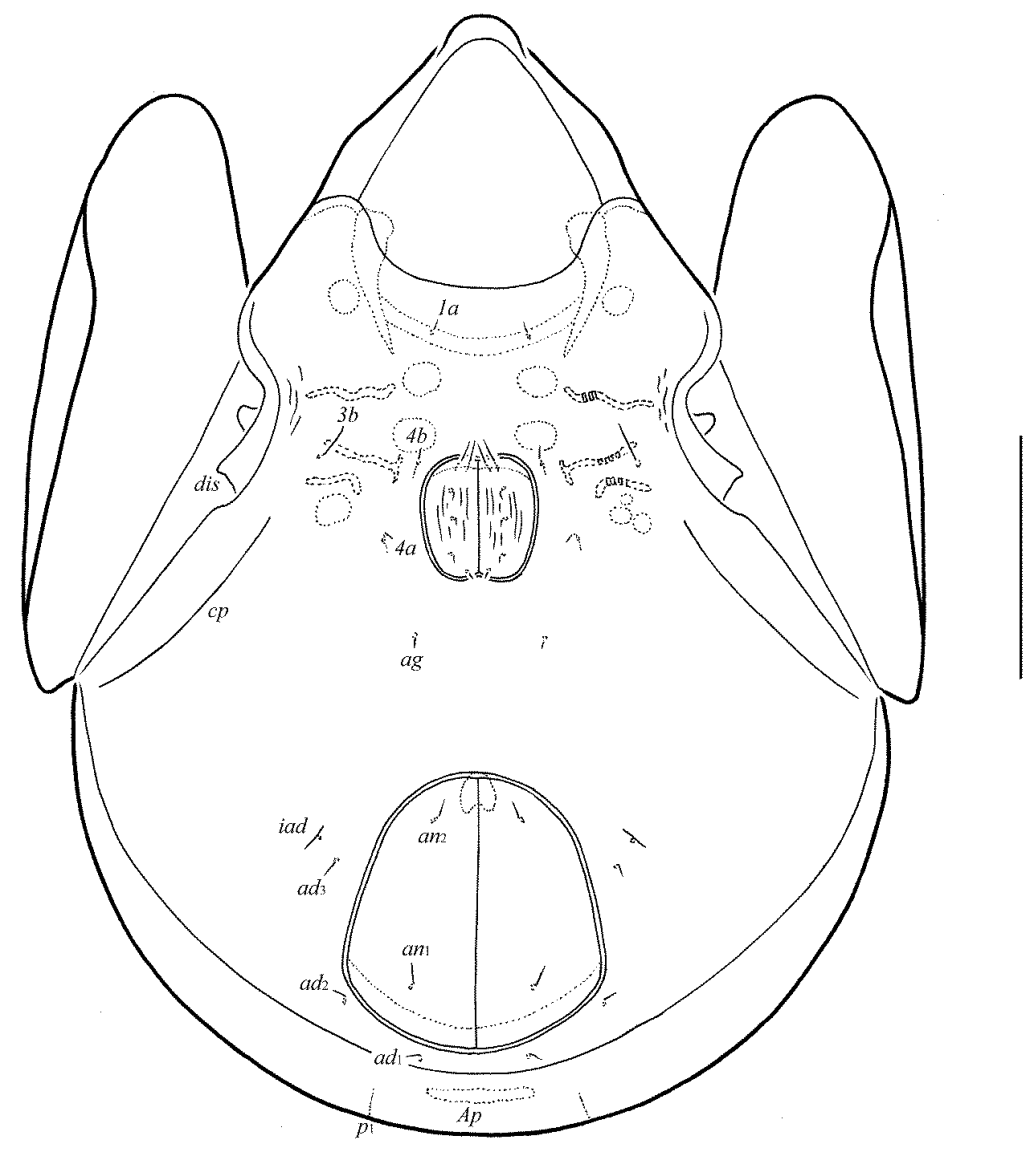
Galumna (Neogalumna) specifica sp. n., adult: ventral view (gnathosoma and legs not shown). Scale bar 100 µm.

**Figures 58–59.**
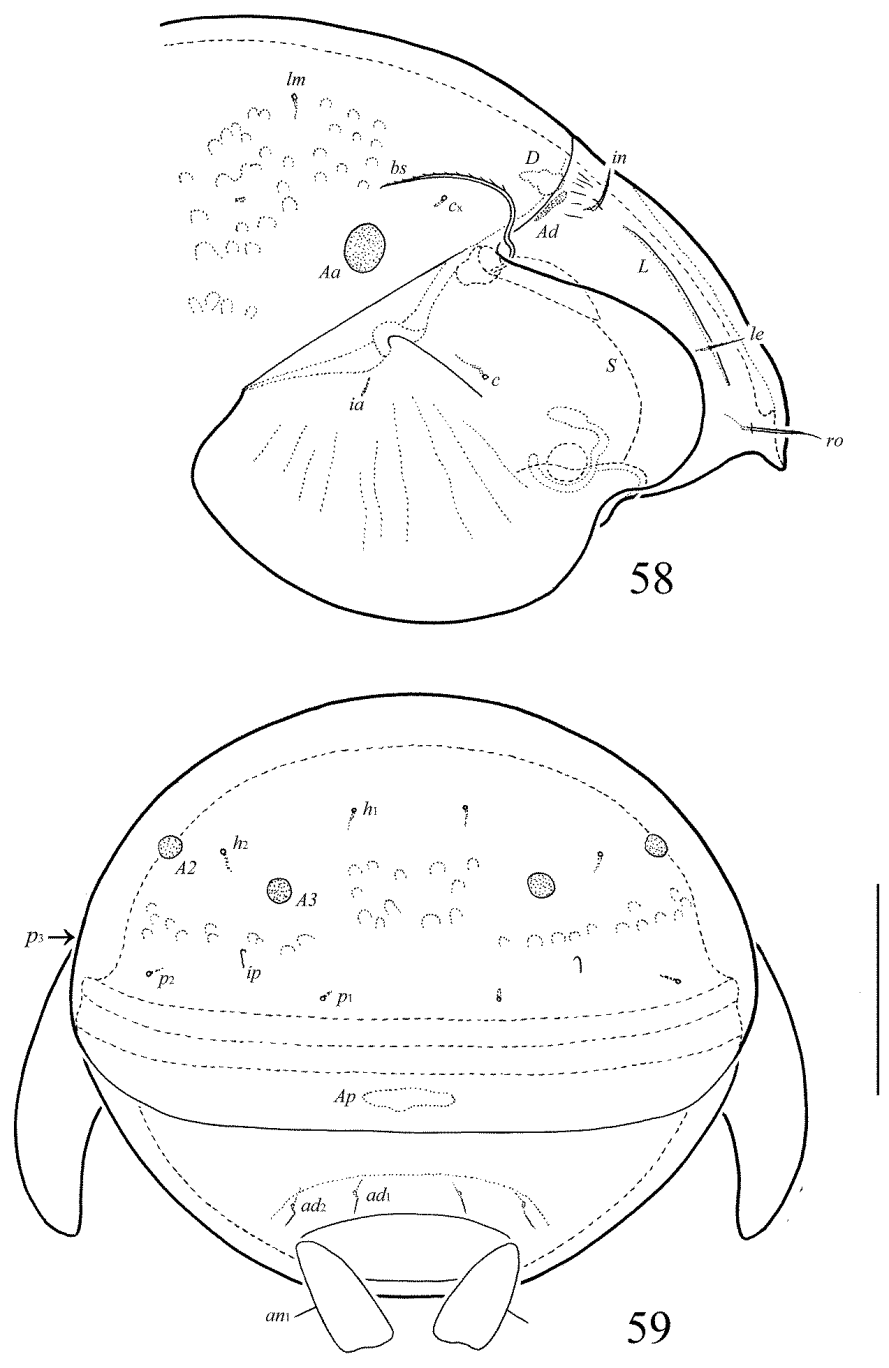
Galumna (Neogalumna) specifica sp. n., adult: **58** anterior part of body, lateral view (gnathosoma and leg I not shown) **59** posterior view. Scale bar 100 µm.

**Figures 60–64.**
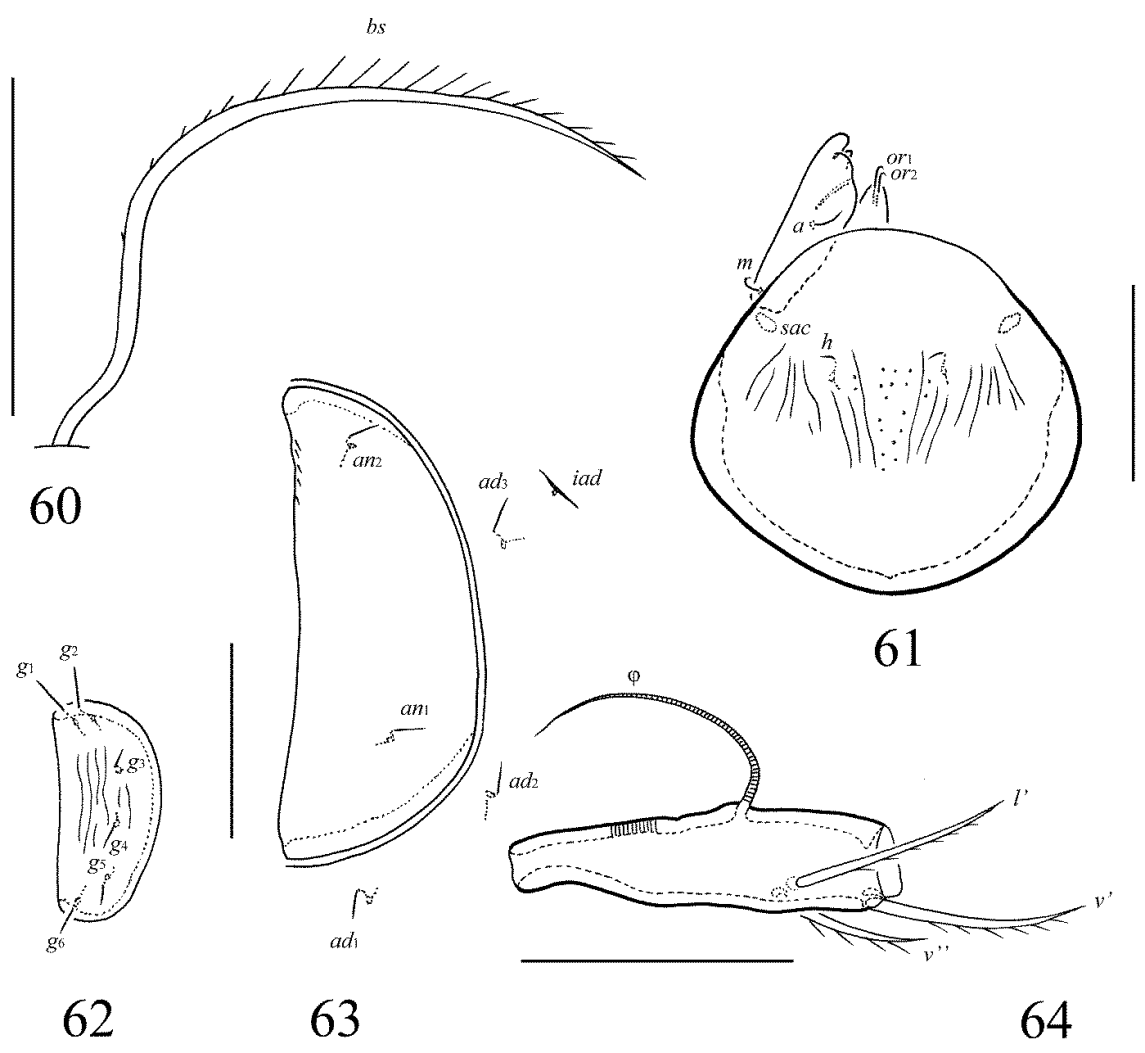
Galumna (Neogalumna) specifica sp. n., adult: **60** bothridial seta **61** subcapitulum (left gena, rutellum and lip not shown), ventral view **62** genital plate, left **63** anal plate, left, and adanal setae **64** tibia of leg IV, left, antiaxial view. Scale bars 50 µm.

#### Material examined.

Holotype (male) and three paratypes (one female and three males): Indonesia, Sumatra, Harapan landscape, secondary rainforest, research site HF4, 02°11'15.2"S, 103°20'33.4"E, 77 m a.s.l., in upper soil layer (0–5 cm). All specimens were collected by Bernhard Klarner (Nov. 2013) and identified and collected to morphospecies level by Dorothee Sandmann.

#### Type deposition.

The holotype is deposited in LIPI (Indonesian Institute of Science) Cibinong, Indonesia; two paratypes are deposited in the collection of the Senckenberg Museum, Görlitz, Germany; one paratype is deposited in the collection of the Tyumen State University Museum of Zoology, Tyumen, Russia.

#### Etymology.

The specific name *specifica* refers to the specific set of notogastral alveoli (*la* absent, *c*_x_ present).

#### Remarks.

Galumna (Neogalumna) specifica sp. n. is morphologically most similar to Galumna (Neogalumna) tolstikovi Ermilov & Anichkin, 2014 from Vietnam (see Ermilov and Anichkin 2014b) in having straight lamellar lines, short prodorsal setae, setiform bothridial setae, setal alveoli *c*_x_ and striate genital plates. However, the new species differs from the latter by larger body size (498–531 × 348–365 vs. 381–415 × 265–298 in Galumna (Neogalumna) tolstikovi), well developed and barbed interlamellar setae (vs. minute in Galumna (Neogalumna) tolstikovi), longest rostral setae on the prodorsum (vs. rostral and lamellar similar in length in Galumna (Neogalumna) tolstikovi), a striate basal part of prodorsum (vs. not striate in in Galumna (Neogalumna) tolstikovi), an elongated postanal porose area (vs. oval in Galumna (Neogalumna) tolstikovi) and the absence of setal alveoli *la* (vs. present in Galumna (Neogalumna) tolstikovi).

### Records

Galumna (Galumna) calva Starý, 1996 (see Starý 1996). Distribution: Australia. New record in the Oriental region.

**Material examined.** Two specimens: Indonesia, Sumatra, Bukit Duabelas landscape, oil palm plantation, research site BO3, 02°04'15.2"S, 102°47'30.6"E, 71 m a.s.l., in forest floor litter material, 15.11.2013 (B. Klarner).

Galumna (Galumna) flabellifera Hammer, 1958 (see Hammer 1958; Aoki 1964, 1982; Mahunka 1978). Distribution: Pantropical and Subtropical regions.

**Material examined.** Two specimens: Indonesia, Sumatra, Bukit Duabelas landscape, oil palm plantation, research site BO5, 02°06'48.9”S, 102°47'44.5”E, 50 m a.s.l., in upper soil layer (0–5 cm). One specimen: same data, but in upper soil layer (0–5 cm). One specimen: Indonesia, Sumatra, Harapan landscape, rubber plantation, research site HR1, 01°54'39.5"S, 103°16'00.1"E, 77 m a.s.l., in upper soil layer (0–5 cm). All specimens were collected by B. Klarner (Nov. 2013) and identified and collected to morphospecies level by Dorothee Sandmann.

Galumna (Galumna) sabahna Mahunka, 1995 (see Mahunka 1995). Distribution: Malaysia. New record in Indonesia.

**Material examined.** Three specimens: Indonesia, Sumatra, Harapan landscape, oil palm plantation, research site HO1, 01°54'35.6"S, 103°15'58.3"E, 81 m a.s.l., in upper soil layer (0–5 cm). Three specimens: Indonesia, Sumatra, Bukit Duabelas landscape, research site BO3, 02°04'15.2"S, 102°47'30.6"E, 71 m a.s.l., in forest floor litter material. One specimen: Indonesia, Sumatra, Harapan landscape, rubber plantation, research site HR1, 01°54'39.5"S, 103°16'00.1"E, 77 m a.s.l., in forest floor litter material. Three specimens: Indonesia, Sumatra, Bukit Duabelas landscape, rubber plantation, research site BR2, 02°05'06.8"S, 102°47'20.7"E, 95 m a.s.l., in upper soil layer (0–5 cm). Four specimens: Indonesia, Sumatra, Bukit Duabelas landscape, rubber plantation, research site HR2, Sumatra, Indonesia, Harapan landscape, S 01°52'44.5’’, E 103°16'28.4’’, rubber plantation, 59 m a.s.l., in forest floor litter material. Three specimens: Indonesia, Sumatra, Bukit Duabelas landscape, rubber plantation, research site HR2, Sumatra, Indonesia, Harapan landscape, S 01°52'44.5’’, E 103°16'28.4’’, rubber plantation, 59 m a.s.l., in upper soil layer (0–5 cm). All specimens were collected by B. Klarner (Nov. 2013) and identified and collected to morphospecies level by Dorothee Sandmann.

## Supplementary Material

XML Treatment for Galumna (Atypicogalumna)

XML Treatment for Galumna (Atypicogalumna) corpuzrarosae

XML Treatment for Galumna (Galumna) bidentatirostris

XML Treatment for Galumna (Galumna) indonesica

XML Treatment for Galumna (Galumna) mikoi

XML Treatment for Galumna (Cosmogalumna) areticulata

XML Treatment for Galumna (Cosmogalumna) sumatrensis

XML Treatment for Galumna (Neogalumna) specifica
